# Structural Diversity of Heteroleptic Cobalt(II) Dicyanamide Coordination Polymers with Substituted Pyrazines and Pyrimidines as Auxiliary Ligands †

**DOI:** 10.3390/molecules30193856

**Published:** 2025-09-23

**Authors:** Joanna Palion-Gazda, Anna Świtlicka, Katarzyna Choroba, Ewa Malicka, Barbara Machura, Agata Trzęsowska-Kruszyńska

**Affiliations:** 1 Institute of Chemistry, University of Silesia, Szkolna 9, 40-006 Katowice, Poland; katarzyna.choroba@us.edu.pl (K.C.); ewa.malicka@us.edu.pl (E.M.); barbara.machura@us.edu.pl (B.M.); 2 Institute of General and Ecological Chemistry, Lodz University of Technology, Zeromskiego 116, 90-924 Lodz, Poland; agata.trzesowska@p.lodz.pl

**Keywords:** coordination polymers, dicyanamide ion, cobalt(II) complexes, X-ray studies

## Abstract

A series of cobalt(II) dicyanamide (dca^−^) coordination polymers with substituted pyrazines (pyz) and pyrimidines (pym) as auxiliary ligands have been synthesized and structurally characterized to investigate the influence of the type and substitution pattern of the auxiliary ligand on the dimensionality and topology of the resulting frameworks. As a result of our studies, 13 novel heteroleptic cobalt(II) dicyanamide coordination polymers were obtained, and their crystal structures were determined by single-crystal X-ray diffraction. Eight of the investigated compounds exhibit a single-chain structure composed of [Co(L_pyz_/_pym_)_2_]^2+^ units bridged via double μ_1,5_–dca ligands. In two complexes, neutral triple-chain topologies were observed, in which double μ_1,5_– and single μ_1,3,5_–dca bridges connect two crystallographically independent cobalt(II) ions, both being six-coordinate in tetragonally elongated octahedral environments. Two- and three-dimensional architectures were confirmed only in the case of Co(II) compounds with 2,6–Me_2_pyz and 4-NH_2_-pym co-ligand, respectively The cobalt(II) complexes described herein have also been compared with dicyanamide-based cobalt(II) systems incorporating pyrazine- and pyrimidine-like ligands. These structural relationships are of high significance for the rational design and synthesis of heteroleptic cobalt(II) dicyanamide systems.

## 1. Introduction

Coordination polymers (CPs), built from metal cations/clusters bridged with organic/inorganic linkers, have attracted considerable attention owing to their intriguing topological structures and interesting properties relevant to potential applications in various fields, such as heterogeneous catalysis, biomedicine, gas storage and separation, magnetism, energy conversion and luminescence [1,2,3,4,5,6,7,8,9,10,11,12]. The functional properties of CPs are fine-tuned by suitable modifications of their chemical composition, final structures and topologies [13,14,15].

**Scheme 1:**
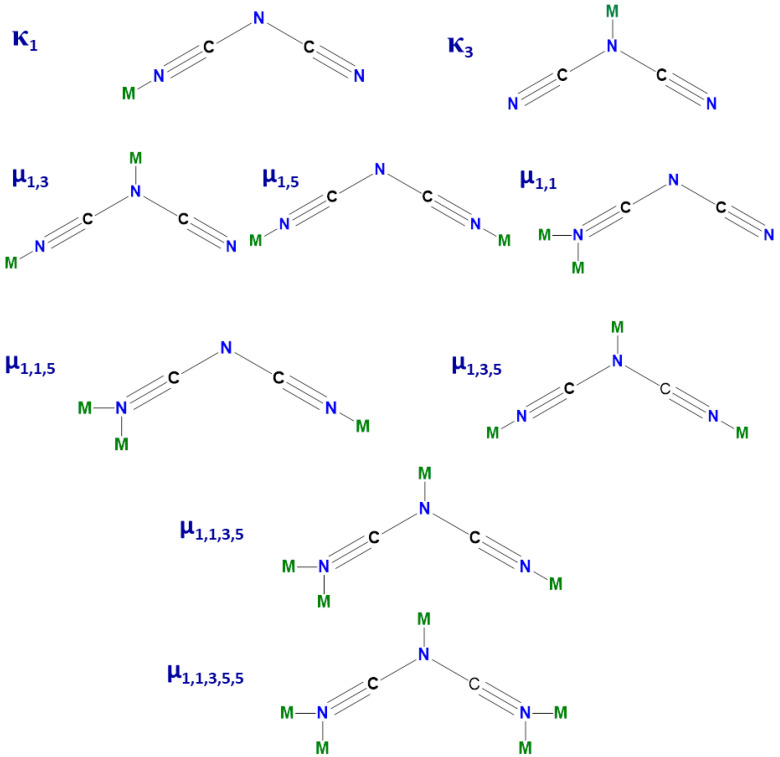
Possible coordination modes of dca^–^ ion. M stands for the metal ion.

Amongst others, intense research efforts have been devoted to CPs based on Co(II) and dicyanamide (dca^–^, N(CN)_2_^−^) ions. The research in this field has been focused on the exploration of structure–magnetic behavior relationships, essential for rational synthesis of molecule-based magnets with pre-defined properties [16,17,18,19,20,21,22]. Regarding structural aspects, the pseudohalide anion dca^–^, with a boomerang shape and three potential nitrogen donor sites (two nitrile and one amide), is a remarkably versatile ligand with multiple coordination modes (Scheme 1), while the cobalt(II) center can exhibit a diversity of stereochemistries, including octahedral, tetrahedral, square-pyramidal, trigonal-bipyramidal, square-planar and pentagonal bipyramidal [23,24].

In the rutile-like three-dimensional (3D) network of α-[Co^II^(dca)_2_] with ferromagnetic behavior, Co(II) ions are octahedrally coordinated to six dca^–^ ligands and each dca^–^ anion is a 3-connector, binding to the metal ion via the nitrile and amide N-donor atoms [25]. The β-form of [Co^II^(dca)_2_], obtained by the depyrimidination of [Co(dca)_2_(pyridine)_2_] and displaying a canted spin antiferromagnetic ordering, was assumed to be isomorphous with β-[Zn(dca)_2_]. It shows a 2D sheet structure and tetrahedral Co(II) ions are linked by μ_1,5_–dca ligands [26]. The network topologies of Co-dca systems and their magnetic properties may be further modulated by incorporation of auxiliary ligands, which occupy certain sites of the metal coordination sphere or act as additional bridges, thus leading to intriguing extended (*n*D, n = 1–3) architectures [16,27,28,29,30,31]. Within this family, there are heteroleptic cobalt(II) dicyanamide coordination polymers with substituted pyrazines as auxiliary ligands, that are 2-aminopyrazine in {[Co(dca)_2_(NH_2_pyz)_2_]·H_2_O}_n_ and HOpyz = 2-hydroxypyrazine in [Co_3_(dca)_6_(HOpyz)_5_(H_2_O)_2_]_n_. Owing to different coordination modes of the pyrazine-based ligands, bis-monodentate for 2-aminopyrazine and monodentate/bridging bis-monodentate for 2-hydroxypyrazine, the obtained polymers differ in the layer structure, which can be described as a typical grid-type array in {[Co(dca)_2_(NH_2_pyz)_2_]·H_2_O}_n_ and a stair-like 2D architecture in [Co_3_(dca)_6_(HOpyz)_5_(H_2_O)_2_]_n_ [32].

**Scheme 2:**
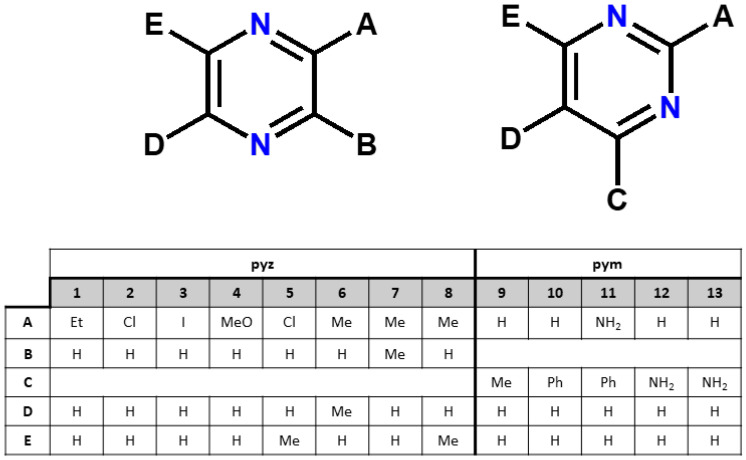
Substituted pyrazines (pyz) and pyrimidines (pym) used to construct of heteroleptic cobalt(II) dicyanamide coordination polymers in the current study.

Although a number of heteroleptic cobalt(II) dicyanamide coordination polymers have been reported so far, the rational design of these materials with precise architectures remains a significant challenge, especially because the self-assembly process is modulated by numerous factors, not only by the building blocks (metal ions and ligands) bearing suitable coordination preferences but also by the conditions of the synthesis, including counterions, solvent polarity, pH value and temperature. The current paper presents the structure correlations for a series of Co(II) dicyanamide coordination polymers with substituted pyrazines (pyz) and pyrimidines (pym) as auxiliary ligands (Scheme 2).

The main emphasis of this contribution is to foresee the impact of the type of substituent and substituent pattern of pyrazine (pyz) and pyrimidine (pym) cores on the coordination mode of the pseudohalide ion and topology of the resulting heteroleptic cobalt(II) dicyanamide framework. The vast majority of obtained systems were found to be 1D coordination polymers, where [Co(L_pyz/pym_)_2_]^2+^ or [Co(H_2_O)_2_]^2+^ units are bridged via double μ_1,5_–dca ligands. [Co_3_(dca)_6_(2–MeOpyz)_4_]*_n_* and {[Co_3_(dca)_6_(2,3–Me_2_pyz)_2_(H_2_O)_2_]·2(2,3–Me_2_pyz)}*_n_* show a triple chain structure, unprecedented in dca-containing cobalt(II) coordination polymers. An intriguing 2D architecture was also confirmed in the case of the heteroleptic cobalt(II) dicyanamide coordination polymer with 2,6–Me_2_pyz as a co-ligand, while the complex [Co(4-NH_2_-pym)(dca)_2_]*_n_* generated a 3D network with bridging dca^–^ and 4-NH_2_-pym ligands. To gain a more complete picture concerning structure correlations for the series of Co(II) dicyanamide coordination polymers with substituted pyrazines (pyz) and pyrimidines (pym) as auxiliary ligands, our results were compared with structural features previously reported for related systems (Tables S1 and S2, respectively).

## 2. Results and Discussion

### 2.1. Synthesis and General Characterization

The heteroleptic cobalt(II) dicyanamide systems, namely [Co(dca)_2_(2–Etpyz)_2_]_n_ (**1**), [Co(dca)_2_(2–Clpyz)_2_]_n_ (**2**), [Co(dca)_2_(2–Ipyz)_2_]_n_ (**3**), [Co_3_(dca)_6_(2–MeOpyz)_4_]_n_ (**4**), [Co(dca)_2_(2–Cl–6–Mepyz)_2_]_n_ (**5**), {[Co(dca)_2_(H_2_O)_2_]·(2,5–Me_2_pyz)}_n_ (**6**) {[Co_3_(dca)_6_(2,3–Me_2_pyz)_2_(H_2_O)_2_]·2(2,3–Me_2_pyz)}_n_ (**7**), [Co(dca)_2_(2,6–Me_2_pyz)]_n_ (**8**), [Co(dca)_2_(4–Mepym)_2_]_n_ (**9**), [Co(dca)_2_(4-Phpym)_2_]_n_ (**10**), [Co(dca)_2_(2-NH_2_–4–Phpym)_2_]_n_ (**11**), [Co(dca)_2_(4–NH_2_pym)_2_]_n_ (**12**) and [Co(dca)_2_(4–NH_2_pym)]_n_ (**13**) were synthesized by the reaction of an aqueous solution of Na[N(CN)_2_] with a methanolic solution of the appropriately substituted pyrazine or pyrimidine and CoCl_2_·6H_2_O or Co(NO_3_)_2_·6H_2_O. The choice of Co(II) salt was found to be pivotal only in the case of 4-amino-pyrimidine, where the synthesis with the use of CoCl_2_ and Co(NO_3_)_2_ resulted in formation of [Co(4-NH_2_-pym)_2_(dca)_2_]_n_ (**12**) and [Co(4-NH_2_-pym)(dca)_2_]_n_ (**13**), respectively.

The presence of dca^–^ ligands in the structures of **1**–**13** is manifested in the FT-IR spectra by very strong intensity peaks [ν_s_(C≡N)] in the region 2177–2190 cm^−1^ and weaker absorptions [ν_as_ + ν_s_(C≡N) and ν_as_(C≡N)] in the range of 2309–2243 cm^−1^ (Figure S1). A displacement of these bands towards higher frequencies relative to those for sodium salt content of the dicyanamide is indicative for the bridging mode of dca^–^ [28,33]. A larger number of the bands corresponding to ν_(C≡N)_ frequencies for **4**, **7** and **8** in comparison to the compounds showing chain structure with double μ_1,5_– bridges (**1**, **2**, **3**, **5** and **6**) is consistent with the presence of two types of dca bridges, that is μ_1,5_ and μ_1,3,5_ coordination modes. The characteristic absorptions of the pyrazine and pyrimidine rings appear as medium or weak bands in the 1440–1682 cm^−1^ and 1457–1641 cm^−1^ regions, respectively. The broad band centered at 3241 cm^−1^ for **6** is attributed to the ν_(O–H)_ stretching. Presence of the amine group attached to the pyrimidine ligand in **11**–**13** is manifested by medium intensity bands attributed to N–H stretching vibrations in the higher energy region 3503–3215 cm^−1^.

The solid reflectance spectra of Co(II) compounds are shown in Figure S2 and electronic spectral data of **1**–**13** are summarized in Table 1. The first three bands of all complexes can be assigned to the spin-allowed electronic transitions for the six-coordinate cobalt(II) complexes in the high-spin octahedral ligand field ^4^T_1g_(F)→^4^T_2g_(F), ^4^T_1g_→^4^A_2g_(F) and ^4^T_1g_(F)→^4^T_1g_(P) [34]. The weaker bands at 16,155 cm^−1^ for **1**, 17,030 cm^−1^ for **3**, 17,182 cm^−1^ for **4**, 16,779 cm^−1^ for **5**, 16,807 cm^−1^ for **6**, 16,807 cm^−1^ for **7** and 16,977 cm^−1^ for **8** (See Figure S2a) may result from spin-forbidden transitions to doublet states derived from 2G or 2H. They cannot be assigned to the second transition ^4^T_1g_→^4^A_2g_(F), because the energy ratio ν_2_/ν_1_ would be out from the 1.9–2.2 range expected for high-spin octahedral Co(II) compounds. The high-energy absorptions in the range 30,030–45,662 cm^−1^ are attributed to n(non-bonding)→π* and π→π* transitions of the organic ligand. For all coordination polymers, the ligand field parameters were calculated from the energy data of the d–d transitions. The parameters B and Dq were calculated on the basis of three frequencies ν_1_, ν_2_ and ν_3_ by using the equations $\mathrm{Dq}\;=\;\frac{1}{10}$(ν_2_ − ν_1_) and $B\;=\;\frac{1}{15}$(ν_3_ + ν_2_ − 3ν_1_). The values of the Dq and B parameters stay in the ranges 1095–911 cm^−1^ and 711–964 cm^−1^, respectively, these values being as expected for cobalt(II) ions in an octahedral environment [34,35,36,37,38]. The nephelauxetic parameter (β < 1), evaluated by the equation β = B/B_0_ (considering B_0_ = 1117 cm^−1^), suggests a significant covalent character in all compounds.

The phase purity of the bulk products **1**–**13** was confirmed by comparing the experimental powder X-ray diffraction (PXRD) patterns with the theoretical ones calculated from the single-crystal data using the Mercury 2.4 program (Figure S3) [39].

### 2.2. Structural Analysis

**Figure 1:**
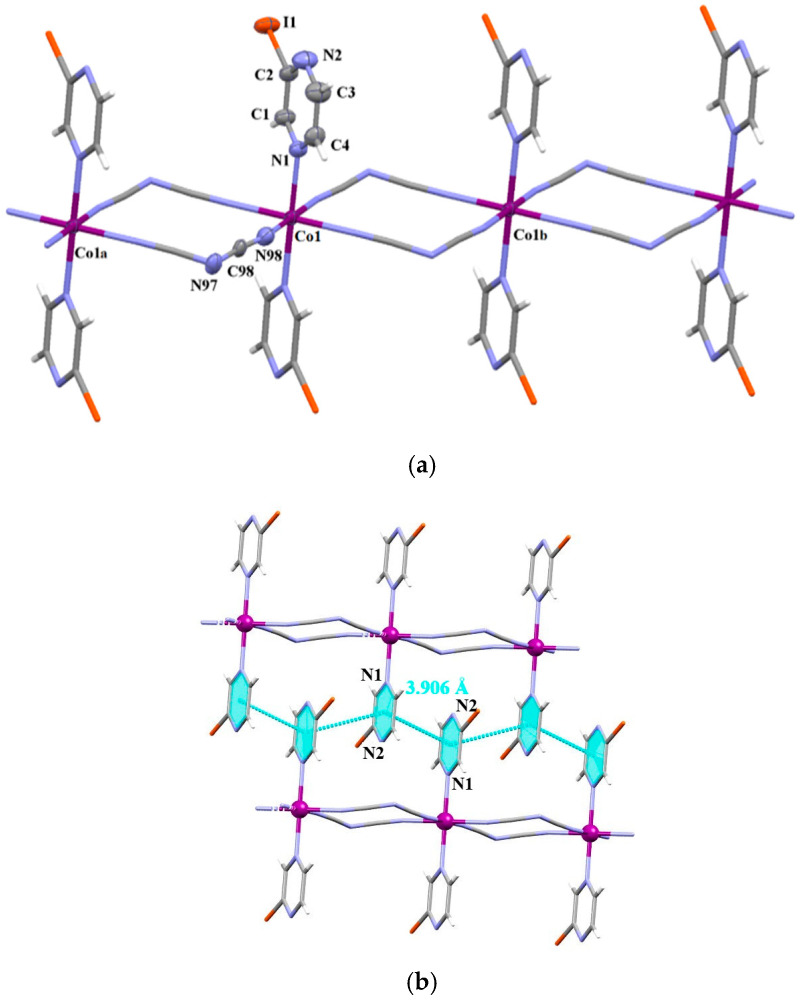
(**a**) View of a fragment of the chain of **3** growing along the crystallographic *b* axis. Displacement ellipsoids are drawn at 50% probability level [Symmetry code: (**a**) = *x*, −1 + *y*, *z*; (**b**) = *x*, 1 + y, z]. (**b**) A view of the supramolecular 2D structure of **3** generated through π–π-type interactions.

**Figure 2:**
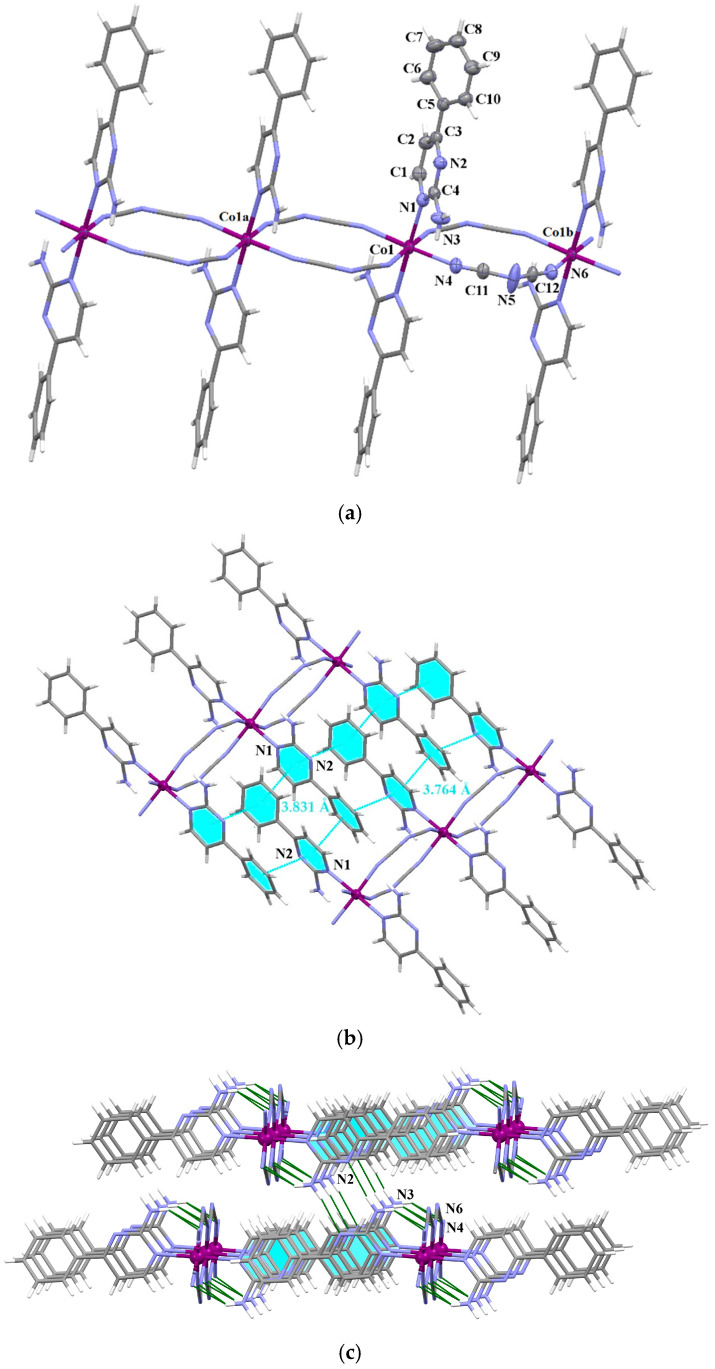
(**a**) The coordination chain of [Co((μ_1,5_–dca)_2_(2-NH_2_–4–C_6_H_5_-pym)_2_]*_n_* (**11**) shown along the crystallographic *b* axis. Displacement ellipsoids are drawn at 50% probability level [symmetry codes: (**a**) = *x*, 1 + *y*, *z*; (**b**) = *x*, −1 + *y*, *z*]; (**b**) Crystal packing of **11** showing π•••π-type interactions; (**c**) the view of fragment of three-dimensional coordination network of **11** formed by π•••π-type interactions and N–H•••N contacts.

**Figure 3:**
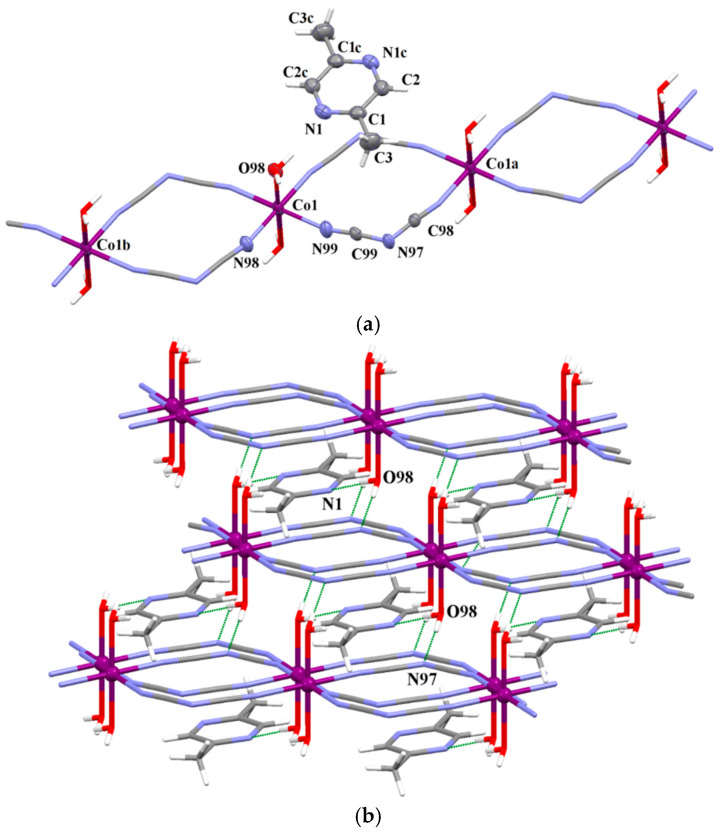
(**a**) View of a fragment of the linear chain [Co(μ–_1,5_–dca)_2_(H_2_O)_2_]_2_ alternating with 2,5-Me_2_pyz molecules in the structure of **6**. Displacement ellipsoids are drawn at 50% probability level [Symmetry code: (**a**) = 1 + *x*, −1 + *y*, *z*; (**b**) = −1 + *x*, 1 + *y*, *z* (**c**) = −*x*, −3 − *y*, −*z*]. (**b**) A view of the 3D crystal packing of **6** generated through O–H**···**N hydrogen bonds.

The single crystal X-ray diffraction studies revealed that **1**–**3**, **5** and **9**–**12** exhibit a chain structure constructed by [Co(L_pyz_/_pym_)_2_]^2+^ units bridged via double μ_1,5_–dca ligands, as shown in Figure 1 (**3**) and Figure 2 (**11**), as well as provided in ESI materials: Figure S4 (**1**), Figure S5 (**2**), Figure S6 (**5**), Figure S7 (**9**), Figure S8 (**10**) and Figure S9 (**12**). The presence of linear coordination chains was also confirmed for compound **6**. In this case, however, double μ_1,5_–dca bridges link [Co(H_2_O)_2_]^2+^ moieties, and water molecules interact with uncoordinated 2,5-Me_2_pyz molecules via hydrogen bonds (Figure 3). Most likely, the large steric demands of the methyl groups of 2,5-Me_2_pyz preclude the coordination of these molecules to the cobalt(II) ion, in contrast to what occurs in **1**–**3**, **5** and **9**–**12**.

In all these systems, the cobalt(II) ions are coordinated to four equatorial μ_1,5_–dca groups via the nitrile nitrogen atoms and a pair of axial pyrazine- (**1**, **2**, **3** and **5**), pyrimidine-based ligands (**9**–**12**) or water molecules (**6**). The octahedral environment of the cobalt(II) ion is tetragonally elongated, as shown by the shortening of Co–Ndca distances relative to the bond lengths Co–N_pyz_ (**1**, **2**, **3** and **5**), Co–N_pym_ (**9**–**12**) and Co–OH_2_ (**6**) (Table 2). The largest elongation of Co–N_pym_ in relation to Co–N_dca_ (~0.09 Å) was found for compound **11** with disubstituted pyrimidine ligand (2-NH_2_–4–C_6_H_5_-pym), while the difference between Co–N_pym_ and Co–N_dca_ distances in **12** is ~0.002 Å. The values of the deviation of the OC-6 geometry for the cobalt(II) ions through the SHAPE analysis are 0.026 (**1**), 0.054 (**2**), 0.054 (**3**), 0.036 (**5**), 0.26 (**6**) 0.023 (**9**), 0.045 (**10**), 0.102 (**11**) and 0.015 (**12**) (to be compared with zero for the ideal octahedron (Figure S10) [40,41]. In all these structures, **1**–**3**, **5**, **6** and **9**–**12**, dca^–^ bridges are angular with the C–N–C angle in the range of 117.6(2)–124.0(2)° and close to linear N–C–N units (173.5(2)–175.3(3)°). The largest angle C–N–C_dca_ accompanied with the longest metal–metal separation through double μ_1,5_–dca bridges was found for **11**. The metal–metal separations through double μ_1,5_–dca bridges in these systems cover the range 7.2579–7.438 Å (see Table 2), and they agree with the distances found in the related systems [Co(µ_1,5_-dca)_2_(2-ampy)_2_]_n_ (7.393 Å; 2-ampy = 2-aminopyridine) [42], [Co(µ_1,5_-dca)_2_(H_2_O)_2_]_n_**·**(hmt)_n_ (7.362 Å; hmt = hexamethylene-tetramine) [43], [Co(µ_1,5_-dca)_2_(imz)_2_]_n_ [7.370 (1) Å; imz = imidazole] [44], [Co((µ_1,5_-dca)_2_(2-pyrrolidone)_2_]_n_ (7.220 Å) [45] and [Co(µ_1,5_-dca)_2_(pydz)_2_]_n_ [7.3409(5) Å; pydz = pyridazine] [31]. More values of bond lengths bond angles around the metal center in structures **1**–**3**, **5**, **6** and **9**–**12** are provided in Tables S3 and S4.

The chains of **1**–**3**, **5**, **10** and **11** are assembled by π–π stacking interactions, into double chains (Table S5 and Figure 1b and Figure 2b and Figures S4–S6 and S10). The coordinated water molecules in the structure of **6** act as hydrogen donors to form O–H•••N hydrogen bonds with the nitrogen atom of the uncoordinated 2,5-dimethylpyrazine molecule [O(98)–H(98)•••N(1) with D⋯A = 2.835(3) Å and D–H⋯A = 171.0°] and amide nitrogen of the dicyanamide bridging ligand of the neighboring chain [O(98)–H(98)•••N(97d) with D•••A = 2.877(3) Å and D–H•••A = 176.0°; symmetry code: (d) = 1 + *x*, *y*, *z*;], leading to formation of a supramolecular 3D network (Figure 2b and Table S5). The amine groups in the structures **11** and **12** act as hydrogen donors to form N–H•••N hydrogen bonds with the amide nitrogen of the dicyanamide bridging. A more detailed structural analysis of **1**–**3**, **5**, **6** and **9**–**12** is available in ESI (Tables S5–S7).

As far as the structures of **4**, **7** and **8** are concerned, two crystallographically independent six-coordinate Co(II) ions [Co(1) and Co(2)] with different coordination environments and the presence of *dca* ligands showing different bridging modes, μ_1,5_– and μ_1,3,5_–*dca*, coexist in them. A comparison of the relevant structural data for **4**, **7** and **8** is shown in Table 3.

**Figure 4:**
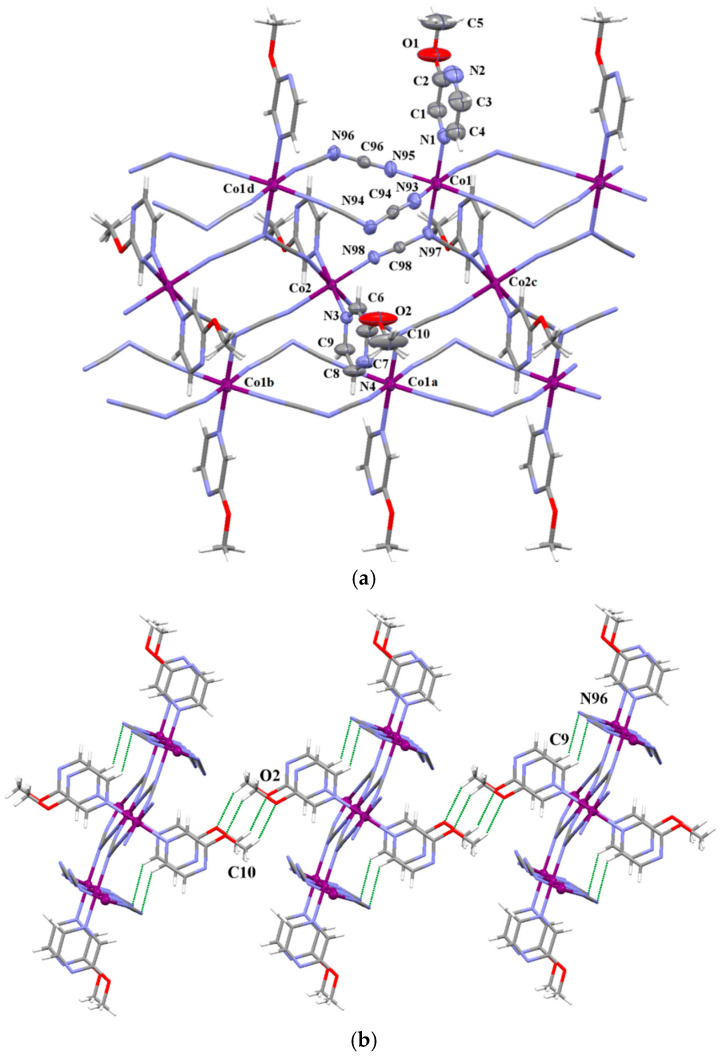
(**a**) Two-dimensional coordination network of **4** formed by μ_1,5_– and μ_1,3,5_–dca bridges [symmetry code: (**a**) = 1 − *x*, 1 − *y*, *z*, (**b**) = 1 − *x*, 1 − *y*, 1 + z (**c**) = *x*, *y*, −1 + *z* and (**d**) = *x*, *y*, 1 + *z*]. Displacement ellipsoids are drawn at the 50% probability level. (**b**) The supramolecular 3D crystal packing in **4** is generated through very weak C–X•••O (X = N and O)-type interactions.

Each Co(2) atom in structure **4** resides at the special Wyckoff position *a* of the *Pbam* space group and its coordination sphere is defined by four nitrile–nitrogen atoms from μ_1,3,5_–*dca* groups [Co(2)–N_dca_ = 2.1206(17) Å] in the equatorial positions and two axially positioned pyrazine-based ligands [Co(2)–L_pyz_ = 2.158(2) Å]. The octahedral coordination sphere of each Co(1) center occupying the *h* special Wyckoff position is defined by four nitrile–nitrogen atoms from four μ_1,5_–*dca* groups in the equatorial plane [Co(1)–N_dca_ = 2.093(2) and 2.104(2) Å] and the amide–nitrogen of a tridentate μ_1,3,5_–*dca* ligand and the nitrogen atom of a 2–MeOpyz molecule in the axial positions [Co(1)-N_dca_ = 2.274(2) and Co(1)-L_pyz_ = 2.152(3) Å] (Figure 4). As a result, each Co(2) center is connected to the four nearest Co(1) atoms through single μ_1,3,5_–dca spacers, whereas each Co(1) is linked in turn to two nearest Co(2) atoms through single μ_1,3,5_–dca groups and two other two Co(1) atoms via double μ_1,5_–dca linkers. Small deviations from the ideal OC-6 geometry of Co1 and Co2 (SHAPE values of 0.109 and 0.027, respectively) occur in **4** (Figure S10). The values of the Co(1)•••Co(1d), Co(1)•••Co(2) and Co(2)•••Co(2c) separations are 7.3175, 6.1415 and 7.3175 Å, respectively. The structure of **4** can be thought as a triple chain (Figure 4a), and to our best knowledge, this topology is unprecedented in dca-containing cobalt(II) coordination polymers. A further examination of the X-ray structure for **4** reveals that triple chains are packed together by very weak C–X•••O (X = N and O)-type interactions (see Table S6) leading to a supramolecular 3D network (Figure 4b).

**Figure 5:**
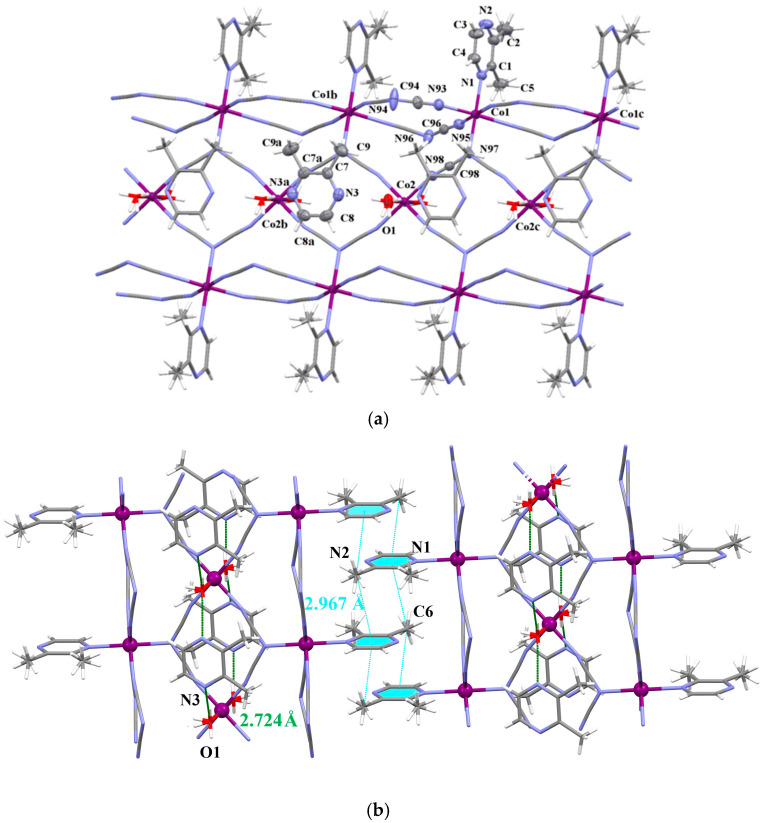
(**a**) View of a fragment of the triple chain of **7** extending along the crystallographic *b* axis together with the uncoordinated 2,3-Me_2_pyz molecules [symmetry code: (**a**) = *x*, 2 − *y*, *z*, (**b**) = *x*, 1 + *y*, z and (**c**) = *x*, −1 + *y*, *z*]. Displacement ellipsoids are drawn at the 50% probability level; (**b**) two-dimensional supramolecular structure assembled by C–H•••π interactions.

The structure of **7** consists of triple neutral chains of formula [Co_3_(dca)_6_(2,3–Me_2_pyz)_2_(H_2_O)_2_]*_n_* alternating with uncoordinated 2,3-dimtehylpyrazine (Figure 5). By analogy to **4**, the Co(1) atoms in **7** are six-coordinated in a somewhat distorted octahedral surrounding which is defined by four nitrile nitrogen atoms of μ_1,5_–*dca* groups in the equatorial sites [Co–N_dca_ = 2.134(3) and 2.092(3) Å] and two nitrogen atoms of μ_1,3,5_–*dca* and 2,3–Me_2_pyz filling the axial positions [Co–N_dca_ = 2.237(3) Å and Co–L_pyz_ = 2.232(3) Å]. In contrast to **4**, the Co(2) ions are in a CoN_4_O_2_ environment, with four equatorial nitrile–nitrogens of μ_1,3,5_–dca ligands [Co–N_dca_ = 2.158(3) Å] and two axial oxygen atoms of water molecules [Co–O_w_ = 2.029(3) Å]. The SHAPE values of the environments of Co(1) and Co(2) with respect to the OC-6 stereochemistry are 0.221 and 0.247, respectively (see Figure S10). Owing to large steric demands of methyl groups, 2,3–Me_2_pyz molecules do not coordinate directly to the Co(2) atoms, but they participate in the formation of hydrogen bonds with water molecules [O(1)–H(1)•••N(3b) with D•••A = 2.724(3) Å and D–H•••A = 167.3°; symmetry code: (b) = *x*, 1 − *y*, *z*]. The Co(1)•••Co(1b) Co(1)•••Co(2) and Co(2)•••Co(2b) separations are 7.4913, 6.1363, 7.4913 Å, respectively. The crystal packing analysis through the Mercury 2.4 program [39] revealed that the neighboring triple chains are assembled by weak C–H•••π stacking interactions into a supramolecular 2D structure (see Figure 5b and Table S7).

**Figure 6:**
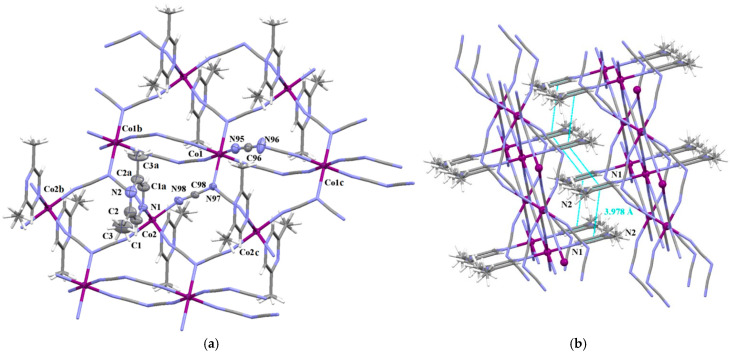
(**a**) View of a fragment of the 2D coordination network of **8** [symmetry code: (**a**) = *x*, 1 − *y*, 1 − *z*, (**b**) = *x*, 1 + *y*, 1 + z (**c**) = *x*, −1 + *y*, *z*]. Displacement ellipsoids are drawn at the 50% probability level. (**b**) A view of the supramolecular 3D network of **8** generated through π–π-type interactions.

The structure of **8** consists of neutral layers of formula [Co(dca)_2_(2,6–Me_2_pyz)]*_n_* which grow parallel to the crystallographic *bc*-plane (Figure 6), and where two crystallographically independent cobalt(II) ions [Co(1) and Co(2)] coexist. The striking difference between the crystal structure of **8** and those of **4** and **7** concerns the coordination sphere of Co(1). In contrast to what occurs in **4** and **7**, the axial positions at the Co(1) atom in **8** are occupied by the amide nitrogens of the μ_1,3,5_–dca bridges. The presence of two μ_1,3,5_–*dca* spacers ligands gives rise to formation of a 2D coordination network which is related to the previously reported for [Co(dca)_2_(pzdo)]*_n_* and [Co(dca)_2_(mpdo)]*_n_* (pzdo = pyrazine 1,4-dioxide and mpdo = 2-methylpyrazine 1,4-dioxide) [46]. Each Co(1) atom, which resides at the special Wyckoff position *a* of the *Cmmm* space group, is connected to the four nearest Co(2) atoms through μ_1,3,5_–dca linkers and also to other two Co(1) atoms via μ_1,5_–dca bridges, while the Co(2) atom is connected to four Co(1) atoms through μ_1,3,5_–*dca* groups. The SHAPE values of the environments of Co(1) and Co(2) with respect to the OC-6 stereochemistry are 0.147 and 0.039, respectively (see Figure S10). The values of the Co(1)•••Co(1b), Co(1)•••Co(2) and Co(2)•••Co(2b) separations are 7.3500, 6.1042 and 7.3500 Å, respectively.

According to the topological analysis performed with the use of ToposPro [47], the coordination polymer framework of **8** can be described as a hxl/Shubnikov 6-*c* plane net {3^6^.4^6^.5^3^}. (Figure S11). The analysis of the crystal packing through the Mercury 2.4 program [39] reveals that the neighboring planes are assembled by π–π stacking interactions into a supramolecular 3D network (Figure 6b and Table S5).

**Figure 7:**
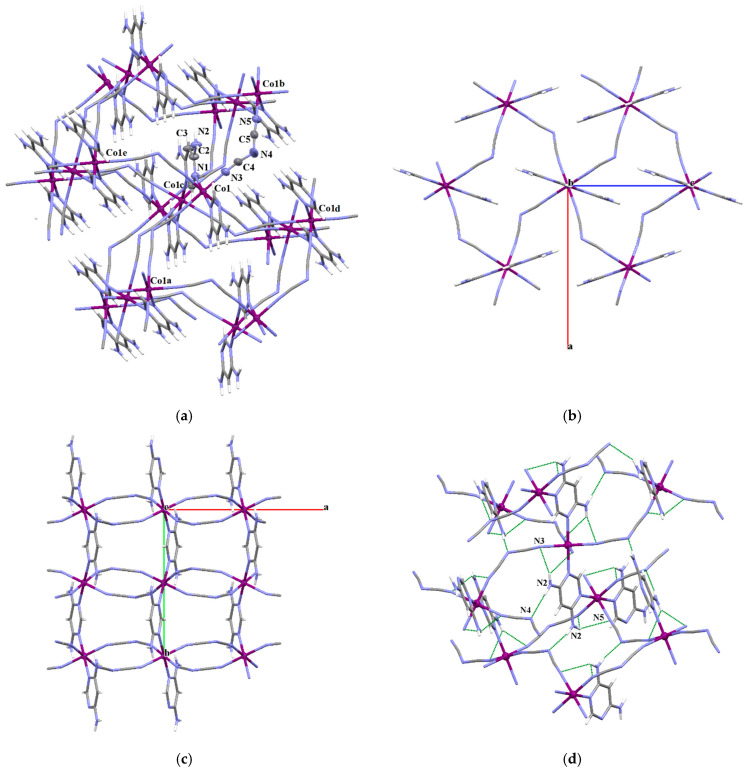
(**a**) Three-dimensional coordination network of **13.** Displacement ellipsoids are drawn at 50% probability level [symmetry codes: (**a**) = −1/2 − *x*, −*y*, −1/2 + *z*; (**b**) = 1/2 − *x*, −*y*, 1/2 + *z*; (**c**) = −*x*, 1/2 + *y*, −*z*; (**d**) = 1/2 − *x*, −*y*, 1/2 + *z*]. (**b**) View of the fragment of 3D coordination polymer of **13** extended (010) plane. (**c**) View of fragment of 3D coordination polymer of **13** extended (001) plane. (**d**) View of the fragment of **13** with N–H•••N hydrogen bonds in the crystal structure.

The compound **13** crystalizes in orthorhombic space group *Pnma* and shows a three-dimensional coordination network (Figure 7). Both dicyanamide and 4-amino-pyrimidine ligands act as two-connecting bridges. The cobalt(II) and *dca*^–^ ions construct a two-dimensional rhombus-grid network parallel to the crystallographic *ac* plane. Each metal ion, located at the center of inversion, is connected to four neighboring metal centers by the single μ_1,5_–*dca* bridges. Similar to the other investigated systems, the dca^–^ spacer is angular with the C–N–C angle of 122.5(4)°, and nearly linear N–C–N units with angles of 172.6(5) and 172.1(5)° (Table S4). The Co–N–C angles in the Co_4_ moiety are 161.2(4) and 162.3(4)°. The intralayer Co•••Co separation through the *dca*^–^ bridge is 8.019 Å, while the metal–metal distances through the diagonals are 12.841 and 9.609 Å, being supportive of rhombic arrangement of Co(II) ions within Co_4_ parallelograms.

The cobalt(II) dicyanamide layers are further linked along the *b* crystallographic axis by the ring N atoms of 4-amino-pyrimidine ligand, forming a 3D coordination network (Figure 7c). The value of the cobalt–cobalt separation across the 4-NH_2_-pym bridge is 5.864 Å. The geometry around the Co(II) ions is best described as an axially elongated octahedron (Table S4), and the Co–N_pym_ is elongated by ~0.05 Å relative to Co–N_dca_.

A further examination of the X-ray structure for **13** reveals that amine groups, equally disordered over two positions due to the constraints of the *Pnma* space group symmetry, act as hydrogen donors to form N–H•••N hydrogen bonds with the amide nitrogen of the dicyanamide bridging ligand, providing further stabilization of the 3D coordination network (Figure 7d).

In summary, we have shown that even minor modifications in the structure of pyrazine- and pyrimidine-derived ligands, as well as in the synthesis parameters, can lead to significant alterations in the topologies of the resulting heteroleptic cobalt(II) dicyanamide systems, as demonstrated in Table 4.

To further demonstrate that the substituent type and substituent pattern of pyrazine and pyrimidine cores may be regarded as an effective tool to control dimensionality and topology of heteroleptic cobalt(II) dicyanamide systems, structural data of **1**–**13** have been compared with those previously reported for related systems (Tables S1 and S2). The use of unsubstituted pyrazine and pyrimidine leads to the formation of three-dimensional networks in which two available donor nitrogen atoms of pyz/or pym and nitrile nitrogens of dca^–^ anions participate in the construction of 3D architectures. In [Co(pyz)(dca)_2_] [48], [Co(pym)(dca)_2_]_n_(PrOH)_n_ [49] and [Co(pym)(dca)_2_]_n_(EtOH)_n_ [49,50], the Co(II) atoms are connected by four µ_1,5_-dca ligands to form 2D sheets, which are further cross-linked by the pyz/pym bridges to give 3D net, likewise in the structure **13**, being isostructural to the reported cobalt(II) dicyanamide with pyrimidines. The three-dimensional structure was also confirmed for {[Co(bpm)(dca)]_2_(ClO_4_)_2_**·** MeOH·2H_2_O}_n_ and {[Co(bpm)(dca)](dca)}_n_, where 2,2′-bipyrimidine (bpm) acts as bis-bidentate ligand and links Co(II) ions into chains, further propagated by µ_1,5_-dca bridges into a 3D framework [51]. Noteworthy, the bpm ligand was found to be able to also form one- and two-dimensional Co(II) polymers in combination with dca^–^ ions, signaling a pivotal role of synthetic conditions in the rational design of heteroleptic cobalt(II) dicyanamide systems [52,53]. A key role of the Co(II) salt used in the synthesis of cobalt(II) dicyanamide with 4-aminopyrazine as an auxiliary ligand was also demonstrated in our studies, which resulted in the formation of two structures differing in dimensionality—[Co(4-NH_2_-pym)_2_(dca)_2_]_n_ (**12,** 1D) and [Co(4-NH_2_-pym)(dca)_2_]_n_ (**13,** 3D). Except for **13**, all cobalt(II) dicyanamide systems with substituted pyrimidines are one-dimensional structures. The presented herein compounds **9**–**12** are linear chains built of [Co(L_pym_)_2_]^2+^ units bridged via double μ_1,5_–dca ligands, while the previously reported [Co(apym)(dca)_2_]n [54] with 2-aminopyrimidine (apym) forms a unique infinite 1D molecular tubes of square cross-section, with the presence of dca ligands bound to Co(II) ions in different coordination modes: μ_1,5_ and μ_1,3,5_. A noticeably larger structural diversity is observed in the family of heteroleptic cobalt(II) dicyanamide systems with substituted pyrazines. The pyrazines functionalized at the 2-position with methyl, amino and hydroxyl groups form 2D networks. The first two systems, {[Co(dca)_2_(Mepyz)_2_]_n_∙H_2_O}_n_ [55] and {[Co(dca)_2_(NH_2_pyz)_2_]·H_2_O}_n_ [32], are typical rhombus-grid sheets based on {Co_4_(μ_1,5_-dca)_4_} units completed with *trans*-positioned R-pyz molecules monodentately coordinated to Co(II) ions, while [Co_3_(dca)_6_(HOpyz)_5_(H_2_O)_2_]_n_ with 2-hydroxypyrazine (OHpyz) [32] exhibits a stair-like 2D network where the intralayer connections are performed by μ_1,5_-dca ions and nitrogen atoms of HOpyz. The introduction of ethyl and halogen groups at the 2-position of the pyrazine ring gives rise to the formation of linear chains with [Co(L_pym_)_2_]^2+^ nodes linked via double μ_1,5_–dca ligands. The same structure type was confirmed for [Co(dca)_2_(quinoxaline)_2_]_n_ [56]. In turn, functionalization at the 2-position with the methoxy group resulted in a unique triple chain structure with two crystallographically independent six-coordinate Co(II) ions and dca ligands showing different bridging modes, μ_1,5_ and μ_1,3,5_. The dimensionality and topology of heteroleptic cobalt(II) dicyanamide systems can also be controlled by the substituent pattern of pyrazine, as demonstrated for {[Co(dca)_2_(H_2_O)_2_]·(2,5–Me_2_pyz)}_n_ (**6**) {[Co_3_(dca)_6_(2,3–Me_2_pyz)_2_(H_2_O)_2_]·2(2,3–Me_2_pyz)}_n_ (**7**), [Co(dca)_2_(2,6–Me_2_pyz)]_n_ (**8**). The presence of bulky methyl groups at the *para* position relative to both pyrazine nitrogen atoms (2,5-Me_2_pyz and 2,3–Me_2_pyz) hinders or even precludes the coordination of these molecules to the cobalt(II) ions (**6** and **7**). Most importantly, structures **7** and **8** are another rare example of heteroleptic cobalt(II) dicyanamide systems with dca ligands showing different bridging modes, μ_1,5_ and μ_1,3,5_. As demonstrated in this work, the coordination mode μ_1,3,5_-dca is significantly less common in the family of cobalt(II) dicyanamide coordination compounds with pyrazine and pyrimidine as auxiliary ligands than μ_1,5_-dca, and its appearance may be evoked by suitable substitution of the pyz ring. Functionalization of the pyz ring with four pyridyl groups gives rise to a versatile polydentate ligand with six potential donor sites—2,3,5,6-tetrakis(2-pyridyl)pyrazine (*tppz*)—able to bind to a metal center in a terminal (bidentate α or γ and tridentate) and bridging [bis(bidentate) α or γ, bidentate α and bis(bidenate) γ and bis(tridentate)] coordination modes [57]. The Cambridge Structural Database [58] survey revealed two 1D cobalt(II) dicyanamide compounds bearing the tppz ligand. Both [Co_2_(tppz)(dca)_4_]ₙ [59] and {[Co_2_(tppz)(dca)_4_]ₙ·CH_3_CN} [60] feature two types of bridging units—the dicyanamide linker and the tppz ligand. The dinuclear [Co_2_(tppz)]^4−^ units of these systems are linked by double µ_1,5_-dca and single µ_1,5_–dca bridges to form chain and ladder-like chain structures. Also, the 1,4,5,8,9,12-Hexaazatriphenylene (HAT) polydentate ligand with three pyz rings was found to bridge Co(II) ions to form the trinuclear {Co_3_(HAT)} units, which are further linked by µ_1,5_-dca ligands to infinite 3D structure [61]. It can be stated that the use of polydentate N-donor auxiliary ligands promotes the formation of polymeric cobalt(II) dicyanamide structures with Co(II) ions linked by both dicyanamide and heterocyclic N-donor ligands.

## 3. Conclusions

In the present contribution, we have synthesized and structurally characterized 13 new dicyanamide-containing cobalt(II) coordination polymers with substituted pyrazine and pyrimidine molecules as neutral co-ligands. The impact of the pyrazine and pyrimidine substituents on the framework of the Co–dca system has been analyzed and discussed relative to the structural data obtained for previously reported heteroleptic cobalt(II) dicyanamide coordination compounds. We evidenced that even small changes in the structure of pyrazine- and pyrimidine-based ligands and synthesis conditions may evoke dramatic changes in the topologies of resulting heteroleptic cobalt(II) dicyanamide systems. The presented findings highlight the critical role of ligand design in controlling the structural diversity of heteroleptic cobalt(II) dicyanamide coordination polymers and provide valuable guidance for the rational design of new functional materials with targeted topologies.

## 4. Materials and Methods

**Materials.** The reagents used to prepare the cobalt(II) complexes were commercially available (Sigma Aldrich, Avantor Poland S.A., Gliwice, Poland) and they were used as received.

**Synthesis of compounds** **1–11**

An aqueous solution (5 cm^3^) of NaN(CN)_2_ (0.075 g, 0.84 mmol) was added dropwise to the mixture of the appropriate pyrimidine derivative (0.84 mmol) and cobalt(II) salt (CoCl_2_∙6H_2_O (0.10 g, 0.42 mmol) or Co(NO_3_)_2_∙6H_2_O (0.12 g, 0.42 mmol) dissolved in methanol (20 cm^3^), and continuously stirred at room temperature for 4 h. The resulting light-pink solution was allowed to evaporate at room temperature and X–ray pink crystals were formed after a few days.

**Synthesis of 12**

An aqueous solution (5 cm^3^) of NaN(CN)_2_ (0.075 g, 0.84 mmol) was added dropwise to the mixture of 4-aminopyrimidine (0.84 mmol) and CoCl_2_∙6H_2_O (0.10 g, 0.42 mmol) dissolved in methanol (20 cm^3^), and continuously stirred at room temperature for 4 h. The resulting light-pink solution was allowed to evaporate at room temperature and X–ray pink crystals were formed after a few days.

**Synthesis of 13**

An aqueous solution (5 cm^3^) of NaN(CN)_2_ (0.075 g, 0.84 mmol) was added dropwise to the mixture of 4-aminopyrimidine (0.04 g, 0.42 mmol) and Co(NO_3_)_2_∙6H_2_O (0.12 g, 0.42 mmol) dissolved in methanol (20 cm^3^), and continuously stirred at room temperature for 4 h. The resulting light-pink solution was allowed to evaporate at room temperature and X–ray pink crystals were formed after a few days.

**[Co(dca)_2_(2–Etpyz)_2_]_n_ (1):** Yield 42%. IR (KBr, cm^−1^): 3090(w), 2970(w), 2313(m), 2242(m), 2177(vs), 1592(w), 1528(w), 1475(w), 1437(w), 1407(w), 1371(m), 1346(sh), 1234(w), 1162(m), 1075(m), 1018(m), 935(w), 849(m), 734(w), 668(w), 527(m), 513(sh), 417(m). UV–Vis (solid state, λ_max_, nm (cm^−1^)): 1077 (9285), 516 (19,379), 490 (20,408), 284 (35,211) and 222 (45,045). Anal. Calc. for C_16_H_16_CoN_10_ (**1**): C, 47.18; H, 3.96; N, 34.39. Found: C, 47.38; H, 3.84; N, 34.59%.

**[Co(dca)_2_(2–Clpyz)_2_]_n_ (2):** Yield 65%. IR (KBr, cm^−1^): 2309(s), 2254(s), 2181(vs) 1521(w), 1455(m), 858(m) and 847(m). UV–Vis-NIR (solid, nm): 1037, 533, 480, 300 and 230. Anal. Calc. for C_12_H_6_Cl_2_CoN_10_ (**2**): C, 34.31; H, 1.44; N, 33.34%. Found: C, 34.69; H, 1.40; N, 33.53%.

**[Co(dca)_2_(2–Ipyz)_2_]_n_ (3):** Yield 36%. IR (KBr, cm^−1^): 3089(w), 2305(m), 2239(m), 2178(vs), 1562(w), 1506(m), 1449(m), 1440(sh), 1374(m), 1347(m), 1279(w), 1144(w), 1115(m), 1055(m), 1010(m), 936(w), 851(w), 739(m), 735(m), 666(w), 633(w), 531(m) and 473(m). UV–Vis (solid state, λ_max_, nm (cm^−1^)): 11,090 (9174), 506 (19,763), 482 (20,747), 296 (33,783) and 226 (44,248). Anal. Calc. for C_12_H_6_CoI_2_N_10_ (**3**): C, 23.90; H, 1.00; N, 23.23. Found: C, 23.60; H, 1.06; N, 23.36%.

**[Co_3_(dca)_6_(2–MeOpyz)_4_]_n_ (4):** Yield 34%. IR (KBr, cm^−1^): 3567(w), 3098(w), 3067(w), 2994(w), 2949(w), 2310(s), 2288(sh), 2266(s), 2245(s), 2180(vs), 1597(m), 1537(s), 1476(m), 1442(m), 1402(s), 1352(s), 1314(s), 1288(sh), 1201(w), 1149(w), 1073(m), 1008(m), 948(w), 908(w), 834(m), 744(w), 683(w), 623(w), 556(w), 534(m), 495(w) and 437(m). UV–Vis (solid state, λ_max_, nm (cm^−1^)): 1117 (8952), 535 (18,691), 497 (20,120), 294 (34,014) and 228 (43,859). Anal. Calc. for C_32_H_28_Co_3_N_26_O_4_ (**4**): C, 37.77; H, 2.77; N, 35.79. Found: C, 37.29; H, 2.37; N, 35.77%.

**[Co(dca)_2_(2–Cl–6–Mepyz)_2_]_n_ (5):** Yield 45%. IR (KBr, cm^−1^): 3108(w), 2310(s), 2254(m), 2182(vs), 1519(m), 1417(w), 1390(w), 1349(m), 1319(w), 1268(w), 1187(m), 1149(w), 1018(m), 947(w), 905(w), 883(w), 733(w), 672(w), 535(m), 506(w), 482(w) and 418(w). UV–Vis (solid state, λ_max_, nm (cm^−1^)): 1086 (9208), 519 (19,268), 486 (20,576), 290 (34,483) and 219 (45,662). Anal. Calc. for C_14_H_10_N_10_CoCl_2_ (**5**): C, 37.52; H, 2.25; N, 31.26. Found: C, 37.08; H, 2.20; N, 31.63%.

**{[Co(dca)_2_(H_2_O)_2_]·(2,5–Me_2_pyz)}_n_ (6):** Yield 83%. IR (KBr, cm^−1^): 3241(br), 2309(s), 2256(m), 2189(vs), 1683(w), 1491(m), 1453(w), 1367(s), 1331(m), 1164(w), 1048(m), 965(w), 929(w), 876(w), 659(w), 510(w) and 418(w). UV–Vis (solid state, λ_max_, nm (cm^−1^)): 1166 (8576), 512 (19,531), 484 (20,661), 273 (36,630) and 227 (44,053). Anal. Calc. for C_10_H_12_CoN_8_O_2_ (**6**): C, 35.83; H, 3.61; N, 33.43. Found: C, 35.49; H, 3.72; N, 33.44%.

**{[Co_3_(dca)_6_(2,3–Me_2_pyz)_2_(H_2_O)_2_]·2(2,3–(Me)_2_pyz)}_n_ (7):** Yield 64%. IR (KBr, cm^−1^): 3089(br), 2310(m), 2263(m), 2244(sh), 2179(vs), 1432(w), 1409(m), 1368(m), 1304(m), 1177(m), 1000(w), 952(w), 889(w), 852(w), 747(w), 672(w), 530(m), 489(w) and 419(w). UV–Vis (solid state, λ_max_, nm (cm^−1^)): 1146 (8726), 525 (19,047), 486 (20,576), 277 (36,101) and 220 (45,455). Anal. Calc. for C_36_H_36_N_26_O_2_Co_3_ (**7**) C, 41.51; H, 3.48; N, 34.96. Found: C, 41.90; H, 3.32; N, 34.43%.

**[Co(dca)_2_(2,6–Me_2_pyz)]_n_ (8):** Yield 66%. IR (KBr, cm^−1^): 3113(w), 2965(w), 2312(m), 2291(m), 2261(s), 2190(vs), 1535(w), 1454(w), 1417(w), 1376(w), 1356(s), 1315(s), 1253(w), 1162(m), 1023(w), 960(w), 943(w), 869(w), 739(w), 682(w), 534(m), 500(m), 477(w) and 419(w). UV–Vis (solid state, λ_max_, nm (cm^−1^)): 1108 (9025), 521 (19,194), 482 (20,747), 279 (35,842) and 224 (43,668). Anal. Calc. for C_10_H_8_CoN_8_ (**8**): C, 40.15; H, 2.70; N, 37.46. Found: C, 40.03; H, 2.75; N, 37.16%.

**[Co(dca)_2_(4-Mepym)_2_]*_n_* (9):** Yield 60%. IR (KBr, cm^−1^): 2305(s) [ν_as_ + ν_s_(C≡N_dca_)], 2246(s) [ν_as_(C≡N_dca_)], 2187(vs) [ν_s_(C≡N_dca_)], 1602(s), 1552(w) and 1485(w) [ν(C=N_Mepym_) and ν(C=C_Mepym_)]; UV–Vis (solid state, λ_max_, nm (cm^−1^)): 1075 (9302), 531 (18,832), 494 (20,243), 297 (33,670), 227 (44,052). Anal. Calc. for C_14_H_12_N_10_Co: C, 44.34; H, 3.19; N, 36.93. Found: C, 44.77; H, 3.34; N, 37.32%. C 44.34% H 3.19% Co 15.54% N 36.93%

**[Co(dca)_2_(4-Phpym)_2_]*_n_* (10):** Yield 65%. IR (KBr, cm^−1^): 2306(s) [ν_as_ + ν_s_(C≡N_dca_)]; 2243(s) [ν_as_(C≡N_dca_)], 2180(vs) [ν_s_(C≡N_dca_)], 1593(s), 1543(m), 1467(m) [ν(C=N_Phpym_) and ν(C=C_Phpym_)]; UV–Vis-NIR (solid, nm): 1038 (9633), 532 (18,796), 502 (19,920), 341 (29,325), 325 (30,769), 265 (33,735), 221 (45,248). Anal. Calc. for C_24_H_16_N_10_Co (**2**): C, 57.26; H, 3.20; N, 27.82%. Found: C, 57.58; H, 3.25; N, 27.46%.

**[Co(dca)_2_(2-NH_2_-4-Phpym)_2_]*_n_* (11):** Yield 55%. IR (KBr, cm^−1^): 3470(m), 3332(m) and 3216(w) [ν(N–H)], 2315(m) [ν_as_ + ν_s_(C≡N_dca_)], 2245(m) [ν_as_(C≡N_dca_)], 2183(s) [ν_s_(C≡N_dca_)], 1632(m), 1599(w), 1549(w) and 1455(m) [ν(C=N_NH2-Phpym_) and ν(C=C_NH2-Phpym_)]; UV–Vis (solid state, λ_max_, nm (cm^−1^)): 1132 (8833), 535 (18,691), 504 (19,481), 392 (25,510), 341 (29,325), 225 (44,444). Anal. Calc. for C_24_H_18_N_12_Co: C, 54.04; H, 3.40; N, 31.51. Found: C, 53.72; H, 3.60; N, 31.11%.

**[Co(dca)_2_(4-NH_2_pym)_2_]*_n_* (12):** Yield 55%. IR (KBr, cm^−1^): 3503(m) and 3375(s) [ν(N–H)], 2282(s) [ν_as_ + ν_s_(C≡N_dca_)], 2246(m) [ν_as_(C≡N_dca_)], 2189(vs) [ν_s_(C≡N_dca_)], 1632(s), 1560(w), 1538(w) and 1499(m) [ν(C=N_NH2pym_) and ν(C=C_NH2pym_)]; UV–Vis (solid state, λ_max_, nm (cm^−1^)): 1131 (8842), 517 (19,342), 495 (20,618), 281 (35,587), 224 (44,642). Anal. Calc. for C_12_H_10_N_12_Co: C, 37.81; H, 2.64; N, 44.09. Found: C, 36.86; H, 2.68; N, 44.51%.

**[Co(dca)_2_(4-NH_2_pym)]*_n_* (13):** Yield 55%. IR (KBr, cm^−1^): 3502(m) and 3374(m) [ν(N–H)], 2285(s) [ν_as_ + ν_s_(C≡N_dca_)], 2274(s) [ν_as_(C≡N_dca_)], 2187(vs) [ν_s_(C≡N_dca_)], 1632(s), 1560(w) and 1541(w) ν(C=N_NH2pym_) and ν(C=C_NH2pym_)]; UV–Vis (solid state, λ_max_, nm (cm^−1^)): 1108 (9025), 534 (18,726), 501 (19,960), 333 (30,030), 290 (34,482), 223 (44,843). Anal. Calc. for C_8_H_4_N_9_Co: C, 33.70; H, 1.41; N, 44.21. Found: C, 33.38; H, 1.49; N, 44.57%.

**Phase purity of the bulk products:** The phase purity of the bulk products **1**–**13** was confirmed by comparing the experimental powder X-ray diffraction (PXRD) patterns with the theoretical ones calculated from the single-crystal data using the Mercury 2.4 program [39].

**Physical techniques.** Elemental analyses were carried out using a Vario EL III CHNOS Elemental Analyzer (Elementar Analysensysteme GmbH, Langenselbold, Germany) (C, H, N). IR spectra were recorded on a Nicolet iS5 spectrophotometer (Thermo Fisher Scientific, Waltham, MA, USA) in the spectral range 4000–400 cm^−1^ with the samples in form of KBr pellets (Figure S1, ESI). The UV–Vis spectra were recorded from solid state samples on spectrophotometer Nicolet Evolution 220 (Thermo Fisher Scientific, Waltham, MA, USA) in the range 190–1100 nm and on spectrophotometer Nicolet iS50 FT-IR (Thermo Fisher Scientific, Waltham, MA, USA) in the range of 700–1500 nm (Figure S2). Powder X-ray diffraction (PXRD) measurements were performed on a PANalytical Empyrean X-ray diffractometer (Malvern Panalytical, Almelo, The Netherlands) using Cu−Kα radiation (λ = 1.5418 Å), in which the X-ray tube was operated at 40 kV and 30 mA ranging from 5 to 50° (Figure S3).

**Crystal structure determination and refinement.** The X-ray diffraction data were collected on single crystals of **1**–**8** by means of an Oxford Diffraction four-circle diffractometer Gemini A Ultra (Rigaku Oxford Diffraction, Abingdon, UK) with Atlas CCD detector (Agilent Technologies, Santa Clara, CA, USA) using graphite monochromated Mo Kα radiation (λ = 0.71073 Å) at room temperature. Diffraction data collection, cell refinement and data reduction were performed using the CrysAlis^Pro^ software [62] The structures were solved by the direct methods using SHELXS97 and refined by full-matrix least-squares on *F*^2^ using SHELXL97 [63]. All the non-hydrogen atoms were refined anisotropically, and hydrogen atoms were placed in calculated positions refined using idealized geometries (riding model) and assigned fixed isotropic displacement parameters, *d*(C–H) = 0.93 Å, *U*_iso_(H) = 1.2 *U*_eq_(C) (for aromatic) and *d*(C–H) = 0.96 Å, *U*_iso_(H) = 1.5 *U*_eq_(C) (for methyl and water). The methyl groups were allowed to rotate about their local threefold axis. Details of the crystalographic data collection, structural determination and refinement for **1**–**13** are given in Tables S8 and S9.

## Figures and Tables

**Table 1:** Electronic spectral data for **1–13**.

Compound	*λ_max_*/nm(cm^−1^)	*Dq*/cm^−1^	*B*/cm^−1^	*Β*
**1**	1077 (9285), 516 (19,379), 490 (20,408), 284 (35,211), 222 (45,045)	1009	795	0.71
**2**	1037 (9643), 533 (18,761), 480 (20,833), 300 (33,333), 230 (43,478)	911	711	0.63
**3**	1090 (9174), 506 (19,763), 482 (20,747), 296 (33,783), 226 (44,248)	1059	866	0.78
**4**	1117 (8952), 535 (18,691), 497 (20,120), 294 (34,014), 228 (43,859)	974	797	0.71
**5**	1086 (9208), 519 (19,268), 486 (20,576), 290 (34,483), 219 (45,662)	1006	814	0.73
**6**	1166 (8576), 512 (19,531), 484 (20,661), 273 (36,630), 227 (44,053)	1095	964	0.86
**7**	1146 (8726), 525 (19,047), 486 (20,576), 277 (36,101), 220 (45,455)	1032	896	0.80
**8**	1108 (9025), 521 (19,194), 482 (20,747), 279 (35,842), 224 (43,668)	1016	858	0.77
**9**	1075 (9302), 531 (18,832), 494 (20,243), 297 (33,670), 227 (44,052)	745	953	0.85
**10**	1038 (9633), 532 (18,796), 502 (19,920) 341 (39,325), 325 (30,769), 265 (33,735), 221 (45,248)	655	916	0.82
**11**	1132 (8833), 535 (18,691), 504 (19,841), 392 (25,510), 341 (29,325), 225 (44,444)	802	986	0.88
**12**	1131 (8842), 517 (19,342), 495 (20,618), 281 (35,587), 224 (44,642)	868	1050	0.94
**13**	1108 (9025), 534 (18,726), 501 (19,960), 333 (30,030), 290 (34,482), 223 (44,843)	774	970	0.87

**Table 2:** Relevant structural data for **1**–**3**, **5**, **6** and **9**–**12**.

Compound	1	2	3	5	6	9	10	11	12
Co–N_dca_ [Å]	2.116(3) 2.120(3)	2.1007(16)	2.103(4)	2.102(4)	2.105(2) 2.113(3)	2.1188(12)	2.111(3) 2.101(3) 2.119(3) 2.126(3)	2.1147(17) 2.1126(16)	2.1260(16)
Co–N_pyz/pym_ [Å]	2.163(3)	2.172(2)	2.178(5)	2.186(5)		2.1541(16)	2.177(3) 2.152(3)	2.2032(15)	2.128(2)
Co–OH_2_					2.1220(19)				
∠(C–N–C)_dca_ [°]	118.9(3)	119.2(2)	118.6(5)	118.9(6)	117.6(2)	119.93(17)	121.0(3) 120.5(3)	124.0(2)	118.0(2)
∠(Co–N–C)_dca_	162.0(3) 159.1(3)	157.07(15)	157.6(4)	160.8(4)	160.8(2) 163.6(2)	157.59(12)	152.5(3) 159.5(3) 162.5(3) 152.0(3)	157.36(16) 156.96(17)	156.57(16)
∠(N–C–N)_dca_	173.7(4) 174.6(3)	174.66(19)	174.5(4)	175.3(5)	175.6(3) 175.3(3)	174.21(15)	172.8(4) 173.1(4) 174.0(3) 174.1(3)	173.5(2) 173.6(2)	174.7(2)
Co•••Co *	7.3512	7.3102	7.3235	7.2579	7.3240	7.386	7.367	7.438	7.330

* Value of the shortest intrachain metal•••metal separation.

**Table 3:** Relevant structural data for **4**, **7** and **8** ^a^.

Compound	4	7	8
Co(1)–N_dca_ [Å]	2.093(2) 2.104(2) 2.274(2)	2.134(3) 2.092(3) 2.237(3)	2.078(4) 2.210(4)
Co(2)–N_dca_ [Å]	2.121(2)	2.158(3)	2.119(3)
Co(1)–N_pyz_ [Å]	2.152(3)	2.232(3)	
Co(2)–N_pyz_ [Å]			2.175(4)
Co–OH_2_ [Å]		2.029(3)	
∠(C–N–C)_1,5–*dca*_ [°]	118.7(3)	121.7(4) 122.3(4)	119.9(6)
∠(Co–N–C)_1,5–*dca*_ [°]	151.9(2) 159.8(2)	158.4(3) 159.2(3)	160.6(4)
∠(N–C–N)_1,5–*dca*_ [°]	174.7(2) 174.8(3)	173.4(4) 174.2(3)	173.9(5)
∠(C–N–C)_1,3,5–*dca*_ [°]	117.0(2)	116.6(3)	117.6(5)
∠(Co–N–C)*_1_*_,3,5–*dca*_ [°]	121.03(12) 163.07(16)	120.62(17) 165.2(2)	121.2(2) 162.7(3)
∠(N–C–N)*_1_*_,3,5–*dca*_ [°]	175.7(2)	175.8(3)	175.4(4)
Co(1)•••Co(2) [Å]	6.1415	6.1363	6.1042
Co(1)•••Co(1d) [Å]	7.3175		
Co(2)•••Co(2c) [Å]	7.3175		
Co(1)•••Co(1b) [Å]		7.4913	7.3500
Co(2)•••Co(2b) [Å]		7.4913	7.3500

^a^ Symmetry codes: (a) = *x*, 1 + *y*, *z* (for **7**); (b) = *x*, 1 + *y*, 1 + *z* (for **8**); (c) = *x*, *y*, −1 + *z;* (d) = *x*, *y*, 1 + *z.*

**Table 4:** Summary table for compounds **1**–**13**.

	1	2	3	4	5	6	7	8	9	10	11	12	13
Space group	*P* $\bar{1}$	*I*2/*m*	*C*2/*m*	*Pbam*	*C*2/*m*	*P* $\bar{1}$	*C*2/*m*	*Cmmm*	*I*2/*m*	*P* $\bar{1}$	*C*2/*m*	*C*2/*m*	*Pnma*
Bridging ligands	μ_1,5_–dca	μ_1,5_–dca	μ_1,5_–dca	μ_1,5_–dca μ_1,3,5_–dca	μ_1,5_–dca	μ_1,5_–dca	μ_1,5_–dca μ_1,3,5_–dca	μ_1,5_–dca μ_1,3,5_–dca	μ_1,5_–dca	μ_1,5_–dca	μ_1,5_–dca	μ_1,5_–dca	μ_1,5_–dca 4-NH_2_-pym
Dimensionality	1D	1D	1D	1D	1D	1D	1D	2D	1D	1D	1D	1D	3D

## Data Availability

Crystallographic data for **1–13** were deposited with the Cambridge Crystallographic Data Center. CCDC Numbers: contain the supplementary crystallographic data for **1–13**. Authors will release the atomic coordinates upon article publication. Copies of this information may be obtained free of charge from the Director, CCDC, 12 Union Road, Cambridge CB2 1EZ, UK (Fax: +44-1223-336033; e-mail: deposit@ccdc.cam.ac.uk or www.ccdc.cam.ac.uk).

## Supplementary Materials

The following supporting information can be downloaded at https://www.mdpi.com/article/10.3390/molecules30193856/s1, Figure S1 IR spectra of **1**–**13**; Figure S2. UV-Vis spectra of powder samples of heteroleptic cobalt(II) dicyanamide systems with pyrazines (a) and pyrimidines (b) co-ligands. Figure S3. The powder XRPD pattern of **1**–**13** (experimental—black) and the simulation of its powder pattern from the crystal structure (red). Figure S4. Coordination chain of **1** shown along the crystallographic a axis. Displacement ellipsoids are drawn at 50% probability level [symmetry codes: (a) = 1 + x, y, z; (b) = −1 + x, y, z]; (b) A view of 2D supramolecular network of 1 generated through π–π type interactions; Figure S5. (a) One-dimensional coordination network of 2 shown along the crystallographic b axis. Displacement ellipsoids are drawn at 50% probability level. (b) View of a fragment of the 2D supramolecular structure of 2 generated through π–π type interactions; Figure S6. One-dimensional coordination network of 5 shown along the crystallographic b axis. Displacement ellipsoids are drawn at 50% probability level. [symmetry code: (a) = x, −1 + y, z; (c) = x, 1 + y, z]. (b) A view of 2D supramolecular structure of 5 generated through π–π type interactions; Figure S7. (a) Coordination chain of **9** shown along the crystallographic b axis. Displacement ellipsoids are drawn at 50% probability level [symmetry codes: (a) = x, 1 + y, z; (b) = x, −1 + y, z]; (b) A view of crystal packing of 9 showing C–H•••π type interactions; (c) Crystal packing of 9 showing the C–H•••N type short contacts; Figure S8. (a) Coordination chain of **10** shown along the crystallographic a axis. Displacement ellipsoids are drawn at 50% probability level [symmetry codes: (a) = 1 + x, y, z; (b) = −1 + x, y, z]; (b) Crystal packing of 10 showing π •••π type interactions; Figure S9. (a) Coordination chain of **12** shown along the crystallographic b axis. Displacement ellipsoids are drawn at 50% probability level [symmetry codes: (a) = x, −1 + y, z; (b) = x, 1 + y, z]; (b) The view of fragment of two-dimensional coordination network of 12 formed by N–H•••N and C–H•••N contacts. Figure S10. The cobalt environment in 1–13 together with shape values (SQ(P)) with respect to the octahedral geometry (OC-6), calculated with SHAPE program; Figure S11. Coordination polymer framework of 8. Table S1: Structural features of Co(II) dicyanamide coordination polymers with substituted pyrazines; Table S2. Structural features of Co(II) dicyanamide coordination polymers with substituted pyrimidines. Table S3. Selected bond lengths (Å) and angles (deg) for **1**−**8**; Table S4. Selected bond lengths (Å) and angles (deg) for **9**−**13**; Table S5. Short π•••π interactions for **1**–**3**, **5**, **8**, **10** and **11**; Table S6. Short intra- and intermolecular contacts detected in structures **2**, **4**, **6** and **7**, **9**–**13**. Table S7. C–H•••Cg(J)(π-ring) interactions for **7** and **9**; Table S8. Crystal data and structure refinement for **1**–**8**; Table S9. Crystal data and structure refinement for **9**–**13**.
