# Supplementary material for: Structural Diversity of Heteroleptic Cobalt(II) Dicyanamide Coordination Polymers with Substituted Pyrazines and Pyrimidines as Auxiliary Ligands
Source: Molecules. 2025 Sep 23;30(19):3856. doi: 10.3390/molecules30193856 (PMC12526020; doi:10.3390/molecules30193856)
Supplement: Supplementary file 1 [file molecules-30-03856-s001.zip › molecules-3866740-supplementary.pdf]

Supporting Information for the manuscript:

Structural diversity of heteroleptic cobalt(II) dicyanamide coordination polymers with substituted pyrazines and pyrimidines as auxiliary ligands

Joanna Palion-Gazda *[1], Anna Świtlicka*[1], Katarzyna Choroba[1], Ewa Malicka[1], Barbara Machura[1], Agata Trzęsowska-Kruszyńska[2]

[1]Institute of Chemistry, University of Silesia, Szkolna 9, 40-006 Katowice, Poland; anna.switlicka@us.edu.pl; joanna.palion-gazda@us.edu.pl

[2] Institute of General and Ecological Chemistry, Lodz University of Technology, Zeromskiego 116, 90-924 Lodz, Poland

Table of Contents

Supporting Information for the manuscript: 1

Table of Contents 2

Table S1. Structural features of Co(II) dicyanamide coordination polymers with substituted pyrazines........ 4

Table S2. Structural features of Co(II) dicyanamide coordination polymers with substituted pyrimidines. 6

Figure S1. The IR spectra of 1-13........ 12

Figure S2. UV-Vis spectra of powder samples of heteroleptic cobalt(II) dicyanamide systems with pyrazines (a) and pyrimidines (b) co-ligands. 12

Figure S3. The powder XRPD pattern of 1-13 (experimental - black) and the simulation of its powder pattern from the crystal structure (red). 16

Figure S4. Coordination chain of 1 shown along the crystallographic *a* axis. Displacement ellipsoids are drawn at 50% probability level [symmetry codes: (a) = 1+*x*, *y*, *z*; (b) = –1+*x*, *y*, *z*]; b) A view of 2D supramolecular network of 1 generated through π–π type interactions. 17

Figure S5. a) One-dimensional coordination network of 2 shown along the crystallographic *b* axis. Displacement ellipsoids are drawn at 50% probability level. b) View of a fragment of the 2D supramolecular structure of 2 generated through π–π type interactions. 18

Figure S6. One-dimensional coordination network of 5 shown along the crystallographic *b* axis. Displacement ellipsoids are drawn at 50% probability level. [symmetry code: (a) = x, –1+*y*, *z*; (c) = *x*, 1+*y*, *z*]. (b) A view of 2D supramolecular structure of 5 generated through π–π type interactions. 19

Figure S7. (a) Coordination chain of 9 shown along the crystallographic *b* axis. Displacement ellipsoids are drawn at 50% probability level [symmetry codes: (a) = *x*, 1+*y*, *z*; (b) = *x*, –1+*y*, *z*]; b) A view of crystal packing of 9 showing C–H•••π type interactions; c) Crystal packing of 9 showing the C–H•••N type short contacts. 20

Figure S8. (a) Coordination chain of 10 shown along the crystallographic *a* axis. Displacement ellipsoids are drawn at 50% probability level [symmetry codes: (a) = 1+*x*, *y*, *z*; (b) = –1+*x*, *y*, *z*]; b) Crystal packing of 10 showing π •••π type interactions. 21

Figure S9. (a) Coordination chain of 12 shown along the crystallographic *b* axis. Displacement ellipsoids are drawn at 50% probability level [symmetry codes: (a) = *x*, –1+*y*, *z*; (b) = *x*, 1+*y*, *z*]; (b) The view of fragment of two-dimensional coordination network of 12 formed by N–H•••N and C–H•••N contacts........ 22

Figure S10. The cobalt environment in 1–13 together with shape values (SQ(P)) with respect to the octahedral geometry (OC-6), calculated with SHAPE program. The shape measure *S*Q(P) is defined as $S_Q(P) = \min\left[(\sum_{i=1}^{n} |\vec{q}_i - \vec{p}_i|^2) / (\sum_{i=1}^{n} |\vec{q}_i - \vec{q}_0|^2) \right] \times 100$, where $\vec{\mathbf{q}_i}$ are *N* vectors that contain the 3*N* Cartesian coordinates of the problem structure Q; $\vec{\mathbf{p}_i}$ contain the coordinates of the

ideal polyhedron P; $\overrightarrow{q_0}$ is the position vector of the geometric centre that is chosen to be the same for the two polyhedra. 24

Figure S11. Coordination polymer framework of 8. 25

Table S3. Selected bond lengths (Å) and angles (deg) for 1−8. 25

Table S4. Selected bond lengths (Å) and angles (deg) for 9−13. 27

Table S5. Short π•••π interactions for 1-3, 5, 8,10 and 11[a] 29

Table S6. Short intra- and intermolecular contacts detected in structures 2, 4, 6 and 7, 9-13.[a] 29

Table S7. C—H•••Cg(J)(π-ring) interactions for 7 and 9.[a] 30

Table S8. Crystal data and structure refinement for 1-8 31

Table S9. Crystal data and structure refinement for 9–13........ 33

Table S1. Structural features of Co(II) dicyanamide coordination polymers with substituted pyrazines.

Refcode	Compound	Bridging ligands	Dimensionality	Co–N$_L$ [Å]	Co–N$_{dca}$ [Å]	Co–N–C$_{dca}$ [°]	C–N–C$_{dca}$ [°]	Co···Co [Å]	Shape value	Ref.
BUKBUE	[Co(pyz)(dca)$_2$]	μ$_{1,5}$-dca;<br>pyz	3D	2.148(2)<br>2.167(2)	2.079(2)<br>2.101(2)<br>2.113(2)<br>2.128(2)	166.3(4)<br>147.1(2)<br>155.9(3)	123.9(3)<br>123.5(3)	8.531*via* single μ$_{1,5}$-dca<br>7.096 *via* pyz	0.088	1
EBATIL	{[Co(dca)$_2$(Mepyz)$_2$]$_n$·H$_2$O}$_n$	μ$_{1,5}$-dca	2D	2.155(1)	2.121(1)<br>2.101(1)	168.3(1)<br>157.0 (1)	125.9(2)<br>125.9(2)	8.319(1) *via* single μ$_{1,5}$-dca	0.032	2
WOZKUU	{[Co(dca)$_2$(NH$_2$pyz)$_2$]·H$_2$O}$_n$	μ$_{1,5}$-dca	2D	2.1712(2)	2.1098(18)<br>2.1194(18)	173.67(17)<br>169.84(18)	122.31(19)	8.211(3) *via* single μ$_{1,5}$-dca	0.052	3
WOZLAB	[Co$_3$(dca)$_6$(HOpyz)$_5$(H$_2$O)$_2$]$_n$	μ$_{1,5}$-dca;<br>HOpyz	2D	Co(1)<br>2.2213(18)<br>2.2024(18)<br>2.1653(16)<br>(Co-O$_{H2O}$)<br><br>Co(2)<br>2.1868(19)	Co(1)<br>2.056(2)<br>2.078(2)<br>2.071(2)<br><br>Co(2)<br>2.114(2)<br>2.073(2)	Co(1)<br>174.0(2)<br>167.2(2)<br>170.2(2)<br><br>Co(2)<br>166.4(2)<br>159.4(2)	123.8(2)<br>126.8(2)<br>120.2(2)	8.475(4) *via* single μ$_{1,5}$-dca<br>8.525(4) *via* single μ$_{1,5}$-dca<br>7.205(6) *via* HOpyz	0.377	3
XANHIE	[Co$_2$(tppz)(dca)$_4$]$_n$	μ$_{1,5}$-dca;<br>tppz	1D	2.156(2)<br>2.114(2)<br>2.140(2)	2.044(2)<br>2.141(2)	168.9(2)<br>152.3(2)	119.1(2)	7.377 *via* double μ$_{1,5}$-dca<br>6.879 *via* tppz	1.770	4
XEPLIP	{[Co$_2$(tppz)(dca)$_4$]$_n$·CH$_3$CN}$_n$	μ$_{1,5}$-dca;<br>tppz	1D	2.078(2)<br>2.213(2)<br>2.220(2)<br>2.005(2)	2.096(2)<br>2.104(2)	170.4(2)<br>172.4(2)	124.6(2)	8.778 *via* single μ$_{1,5}$-dca<br>6.916 *via* tppz	1.981	5
MOSDUT	[Co$_3$(HAT)[N(CN)$_2$]$_6$(OH$_2$)$_2$	μ$_{1,5}$-dca;<br>HAT	3D	Co(1)<br>2.215(6)<br>2.156(7)<br>Co(2)<br>2.160(6)<br>2.213(9)<br>Co(3)<br>2.182(9)<br>2.186(6)	Co(1)<br>2.055(7)<br>2.06(1)<br>2.049(8)<br>2.120(6) (Co-O$_{H2O}$)<br>Co(2)<br>2.05(1)<br>2.136(8) (Co-	Co(1)<br>171.7(7)<br>167.7(8)<br>174.7(9)<br>Co(2)<br>170.8(9)<br>Co(3)<br>153.2(9)	118.0(9)<br>122.3(2)<br>124.1(1)	7.760(2) *via* single μ$_{1,5}$-dca<br>7.078(2) *via* HAT	0.898<br>1.220<br>0.970	6

					O_{H2O})<br>Co(3)<br>2.088(9)<br>2.03(1)					
PAHVUQ	$[Co(dca)_2(qui)_2]_n$	$\mu_{1,5}$-dca	1D	2.257(2)	2.098(3)<br>2.104(3)	160.5(2)<br>159.8(1)	119.9(3)	7.374(1)	0.027	7

pyz – pyrazine; **Mepyz** –2-methylpyrazine; **NH_2pyz** – 2-aminopyrazine; **HOpyz** – 2-hydroxypyrazine; **tppz** – tetra-2-pyridylpyrazine; **HAT** –1,4,5,8,9,12-Hexa-azatriphenylene; **qui**- quinoxaline

References:

1. Jensen, P.; Batten, S.R.; Moubaraki, B.; Murray, K.S. Synthesis, structural isomerism, and magnetism of the coordination polymers $[M(dca)_2pyz]$ (M = Mn, Fe, Co, Ni, and Zn; dca = dicyanamide $(N(CN)_2^-)$; pyz = pyrazine). *J. Solid State Chem.* **2001**, *159*, 352–361.
2. Kutasi, A.M.; Harris, A.R.; Batten, S.R.; Moubaraki, B.; Murray, K.S. Coordination polymers of dicyanamide and methylpyrazine: Syntheses, structures, and magnetic properties. *Cryst. Growth Des.* **2004**, *4*, 605–610.
3. Palion-Gazda, J.; Świtlicka, A.; Choroba, K.; Machura, B.; Kruszynski, R.; Julve, M. Influence of the pyrazine substituent on the structure and magnetic properties of dicyanamide-bridged cobalt(II) complexes. *Dalton Trans.* **2019**, *48*, 17266–17280.
4. Hsu, G.-Y.; Chen, C.-W.; Cheng, S.C.; Lin, S.-H.; Wei, H.-H.; Lee, C.-J. Structure and magnetic properties of one-dimensional metal complexes constructed from alternating dicyanamide linked through binuclear metal tetra-2-pyridylpyrazine subunits. *Polyhedron* **2005**, *24*, 487–494.
5. Luo, J.; Qiu, L.; Liu, B.; Zhang, X.; Yang, F.; Cui, L. Synthesis, structure, and magnetic properties of two cobalt(II) dicyanamide (dca) complexes with heterocyclic nitrogen donors tetra(2-pyridyl)pyrazine (tppz) and 2,4,6-tri(2-pyridyl)-1,3,5-triazine (tptz): $[Co_2(tppz)(dca)_4]\cdot CH_3CN$ and Co(tptz)(dca)(H_2O). *Chin. J. Chem.* **2012**, *30*, 522–528.
6. Marshall, S.R.; Rheingold, A. L.; Dawe, L. N.; Shum, W. W.; Kitamura, C.; Miller, J. S. Corner Sharing Tetrahedral Network in $Co_3(HAT)[N(CN)_2]_6(OH_2)_2$ (HAT) 1,4,5,8,9,12-Hexaazatriphenylene). *Inorg. Chem.* **2002**, *41*, 3599−3601.
7. Luo, J.; Liu, B.S. Zhou, X.G.; Weng, L.H; Li, Y.R.; We, H.X. catena-poly[bis[[quinoxaline-*κN*)-cobalt(II)]-di-μ-dicyanamido-$\kappa^2 N^1:N^5$] and catena-poly[bis[[quinoxaline-*κN*)-copper(II)]-di-μ-dicyanamido-$\kappa^2 N^1:N^5$]. *Acta Cryst.,Sect.C:Cryst. Struct. Commun.* **2004**, *60*, m520.

Table S2. Structural features of Co(II) dicyanamide coordination polymers with substituted pyrimidines.

Refcode	Compound	Bridging ligands	Dimensionality	Co–N_L [Å]	Co–N_{dca} [Å]	Co–N–C_{dca} [°]	C–N–C_{dca}	Co···Co [Å]	Shape value	Ref.
MEKLUJ	$[Co(apym)(dca)_2]n$	$\mu_{1,5}$-dca<br>$\mu_{1,3,5}$-dca	1D	2.149(2)	2.110(1)<br>2.089(1)<br>2.224(2)	$\mu_{1,5}$-dca<br>162.69(12)<br>162.06(12)<br>$\mu_{1,3,5}$-dca<br>120.70(9)	117.71(17)	6.118 *via* $\mu_{1,3,5}$-dca	0.145	1
QEHLEU	$[Co(bpm)(H_2O)(dca)_2]_n$	$\mu_{1,5}$-dca	1D	2.151(3)<br>2.124(4)<br>(Co-O_{H2O})	2.092(4)	161.8 (6)<br>150.8(7)	124.9(9)	8.649 *via* $\mu_{1,3,5}$-dca	0.342	2
QEFQEX	$[Co_2(bpm)(dca)_4]_n·H_2O\}_n$	$\mu_{1,5}$-dca;<br>bpm	2D	2.165(3)<br>2.167(3)	double $\mu_{1,5}$-dca<br>2.075(3)<br>2.096(3)<br>single $\mu_{1,5}$-dca<br>2.114(4)<br>2.102(4)	double $\mu_{1,5}$-dca<br>158.6(3)<br>161.2(3) single $\mu_{1,5}$-dca<br>164.5(3)<br>158.8(3)	117.9<br>120.9(4)	7.220(2) *via* double $\mu_{1,5}$-dca<br>8.288(2) *via* single $\mu_{1,5}$-dca<br>5.766(2) *via* pym	0.471	2
QEFQEX01	$\{[Co_2(bpm)(dca)_4]·H_2O\}_n$	$\mu_{1,5}$-dca;<br>bpm	2D	2.165(2)<br>2.165(2)	double $\mu_{1,5}$-dca<br>2.098(3)<br>2.079(3)<br>single $\mu_{1,5}$-dca<br>2.103(3)<br>2.103(3)	double $\mu_{1,5}$-dca<br>158.3(3)<br>160.1(3) single $\mu_{1,5}$-dca<br>160.2(3)<br>163.8(3)	117.3<br>114.1	7.201(1) *via* double $\mu_{1,5}$-dca<br>8.299(1) *via* single $\mu_{1,5}$-dca<br><br>5.771(1) *via* pym	0.442	3
LAFKUZ	$[Co(pym)(dca)_2]_n(PrOH)_n$	$\mu_{1,5}$-dca;<br>pym	3D	*						4
UFIKEZ	$[Co(pym)(dca)_2]_n(EtOH)_n$	$\mu_{1,5}$-dca;<br>py	3D	2.154(4)	2.103(4)<br>2.105(4)	*			–	5
UFIKEZ01	$[Co(pym)(dca)_2]_n(EtOH)_n$	$\mu_{1,5}$-dca;<br>pym	3D	2.154(4)	2.103(4)<br>2.105(4)	*		7.9091(2) *via* dca<br>5.9634(2) *via*	–	4

								pym		
VEYREX	{[Co(bpm)(dca)]$_2$(ClO$_4$)$_2$·MeOH·2H$_2$O}$_n$	μ$_{1,5}$-dca; bpm	3D	2.145(5) 2.163(3) 2.139(3) 2.184(4)	2.077(5) 2.044(4)	162.9(5) 170.8(4)	123.1(5)	7.8407(8) *via* dca 5.6960(8) *via* pym	0.982	6
VEYRIB	{[Co(bpm)(dca)](dca)}$_n$	μ$_{1,5}$-dca; bpm	3D	2.137(2) 2.172(2) 2.145(2) 2.187(2)	2.056(3) 2.074(3)	152.8(2) 171.3(2)	124.6(3)	8.2321(4) *via* dca 5.7345(4) *via* bpm	0.840	6

pym – pyrimidine; **apym** – 2-aminopyrimidine; **bpm** - 2,2′-bipyrimidine; **PrOH**- propanol, **EtOH**- ethanol; **MeOH**- methanol

* - no cif file available

References:

1. Jensen, P.; Batten, S.R.; Moubaraki, B.; Murray, K.S. Infinite molecular tubes: Structure and magnetism of M(dca)$_2$(apym) [M = Co, Ni, apym = 2-aminopyrimidine, dca = dicyanamide, N(CN)$_2$–]. *Chem. Commun.* **2000**, 793–794.

2. Marshall, S.R.; Incarvito, C.D.; Manson, J.L.; Rheingold, A.L.; Miller, J.S. Synthesis, structure, and magnetic properties of Co$_2${[N(CN)$_2$]$_4$bpym}·H$_2$O and M{[N(CN)$_2$]$_2$bpym}·H$_2$O (M = Mn, Fe, Co; bpym = 2,2′-bipyrimidine). *Inorg. Chem.* **2000**, *39*, 1969–1973.

3. Martin, S.; Barandika, M.G.; Cortes, R.; Ruiz de Larramendi, J.I.; Urtiaga, M.K.; Lezama, L.; Arriortua, M.I.; Rojo, T. The 2D and 3D compounds [M$_2$bpm(dca)$_4$]·nH$_2$O (M = Ni, Zn; bpm = bipyrimidine; dca = dicyanamide; n = 0, 1): Magnetic properties. *Eur. J. Inorg. Chem.* **2001**, 2107–2112.

4. Takagami, N.; Ishida, T.; Nogami, T. Single-crystal magnetic study on guest-tunable weak ferromagnets M{N(CN)$_2$}$_2$(pyrimidine) (M = Fe, Co). *Bull. Chem. Soc. Jpn.* **2004**, *77*, 1125–1134.

5. Kusaka, T.; Ishida, T.; Hashizume, D.; Iwasaki, F.; Nogami, T. Low-temperature magnets M[N(CN)$_2$]$_2$(pyrimidine) (M = Fe and Co) with a 3-D network. *Chem. Lett.* **2000**, 1146–1147.

6. Chen, C.W.; Lee, C.J. Two new 3D networks Co(II) complexes constructed via the bridging dicyanamide and 2,2-bipyrimidine ligands: Structures and magnetic properties. *J. Chin. Chem. Soc.* **2006**, *53*, 1291–1296.

1

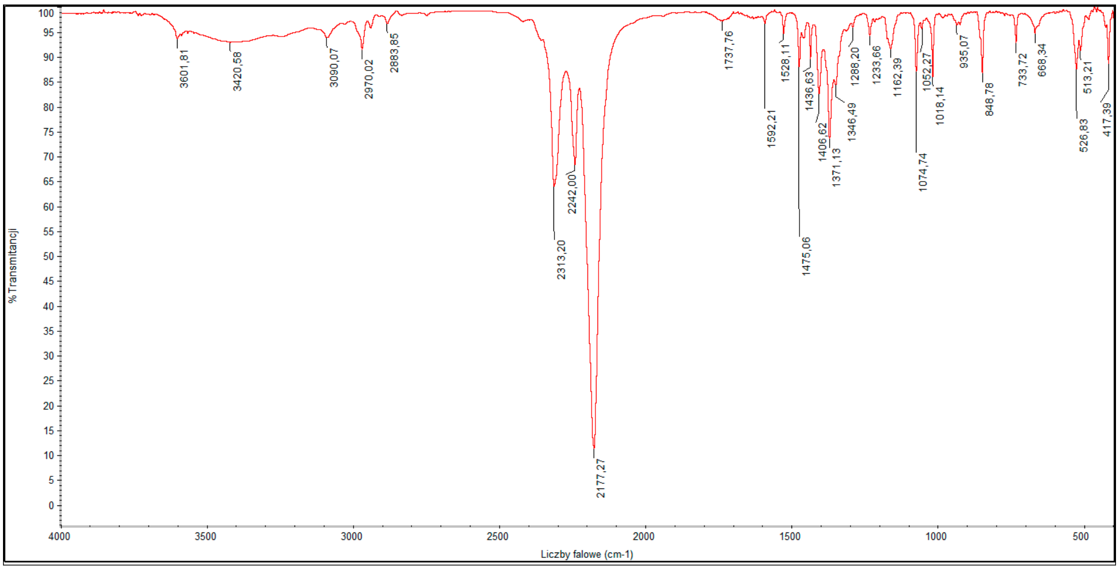
% Transmitancji
Liczby falowe (cm-1)
3601,81
3420,58
3090,07
2970,02
2883,85
2313,20
2242,00
2177,27
1737,76
1592,21
1528,11
1475,06
1436,63
1406,62
1371,13
1346,49
1288,20
1233,66
1162,39
1074,74
1052,27
1018,14
935,07
848,78
733,72
668,34
526,83
513,21
417,39

2

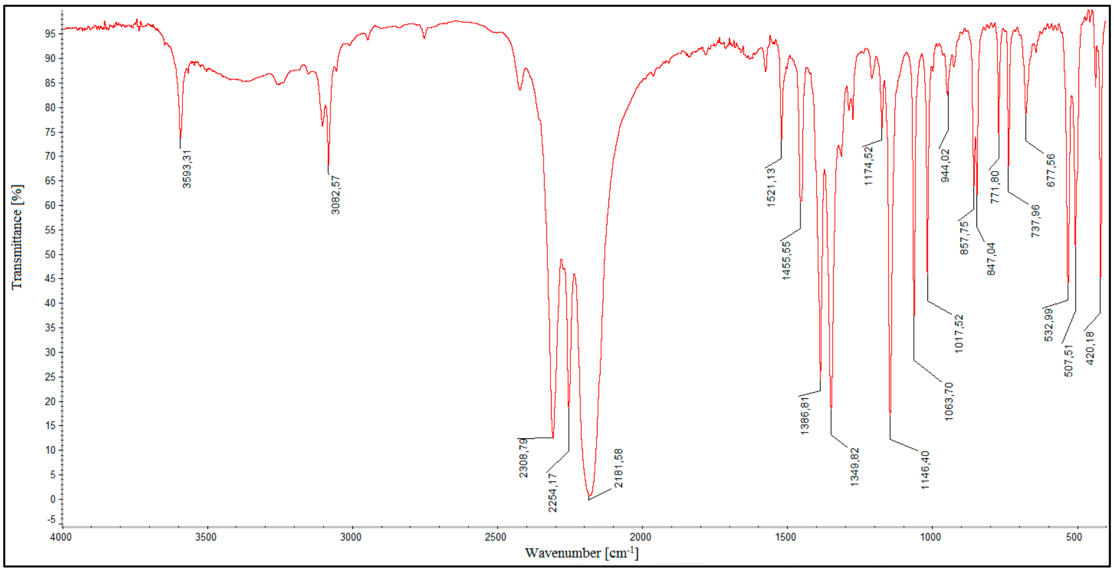
Transmittance [%]
Wavenumber [cm-1]
3593,31
3082,57
2308,79
2254,17
2181,58
1521,13
1455,55
1386,81
1349,82
1174,52
1146,40
1063,70
1017,52
944,02
857,75
847,04
771,80
737,96
677,56
532,99
507,51
420,18

3

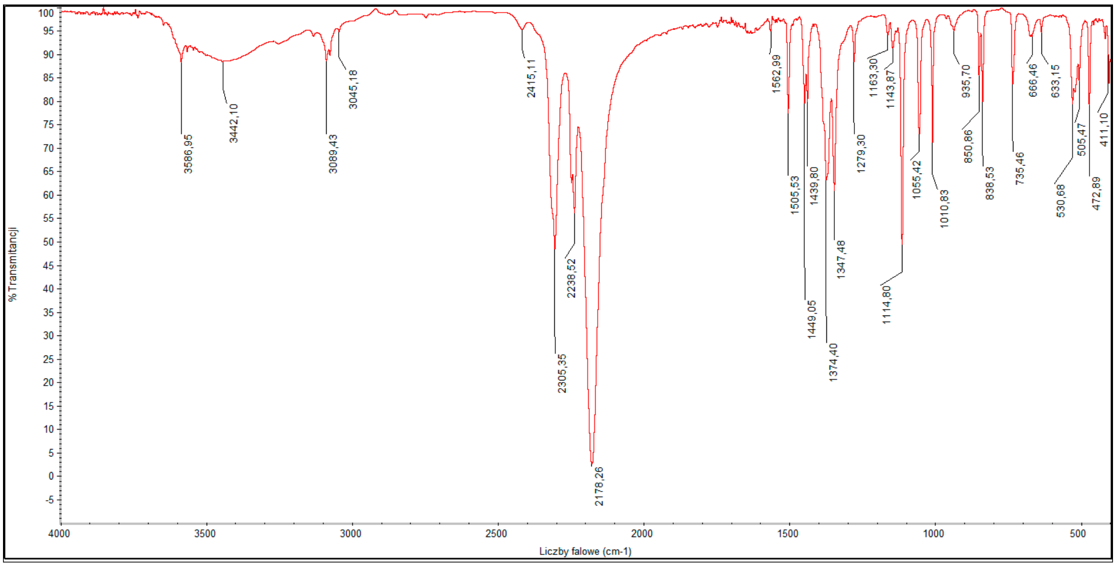
% Transmitancji
Liczby falowe (cm-1)
3586,95
3442,10
3089,43
3045,18
2415,11
2305,35
2238,52
2178,26
1562,99
1505,53
1449,05
1439,80
1374,40
1347,48
1279,30
1163,30
1143,87
1114,80
1055,42
1010,83
935,70
850,86
838,53
735,46
666,46
633,15
530,68
505,47
472,89
411,10

4

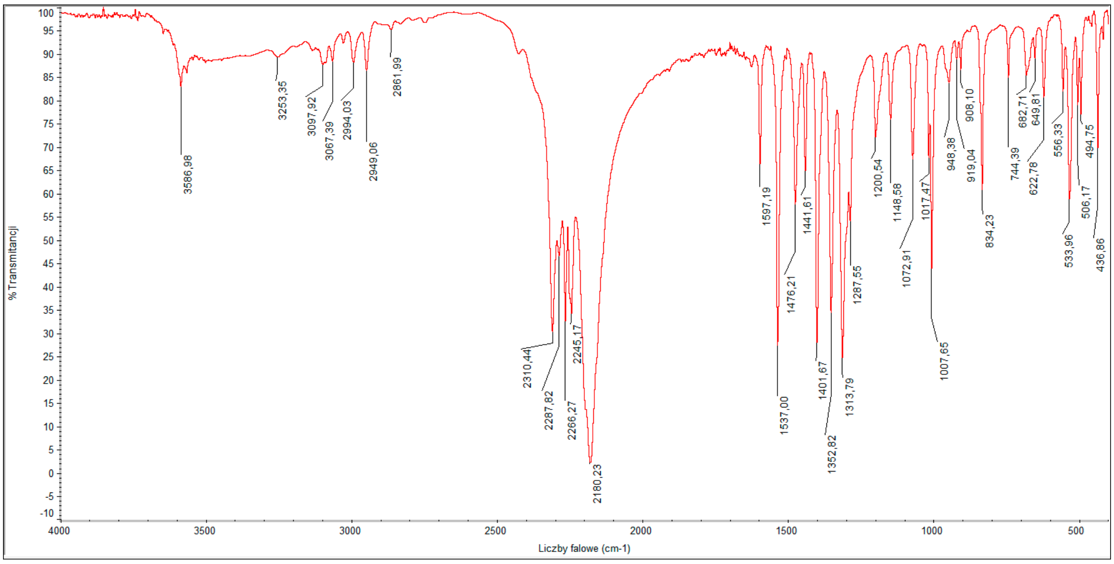
% Transmitancji
Liczby falowe (cm-1)
100
95
90
85
80
75
70
65
60
55
50
45
40
35
30
25
20
15
10
5
0
-5
-10
4000
3500
3000
2500
2000
1500
1000
500
3586,98
3253,35
3097,92
3067,39
2994,03
2949,06
2861,99
2310,44
2287,82
2266,27
2245,17
2180,23
1597,19
1537,00
1476,21
1441,61
1401,67
1352,82
1313,79
1287,55
1200,54
1148,58
1072,91
1017,47
1007,65
948,38
919,04
908,10
834,23
744,39
682,71
649,81
622,78
556,33
533,96
506,17
494,75
436,86

5

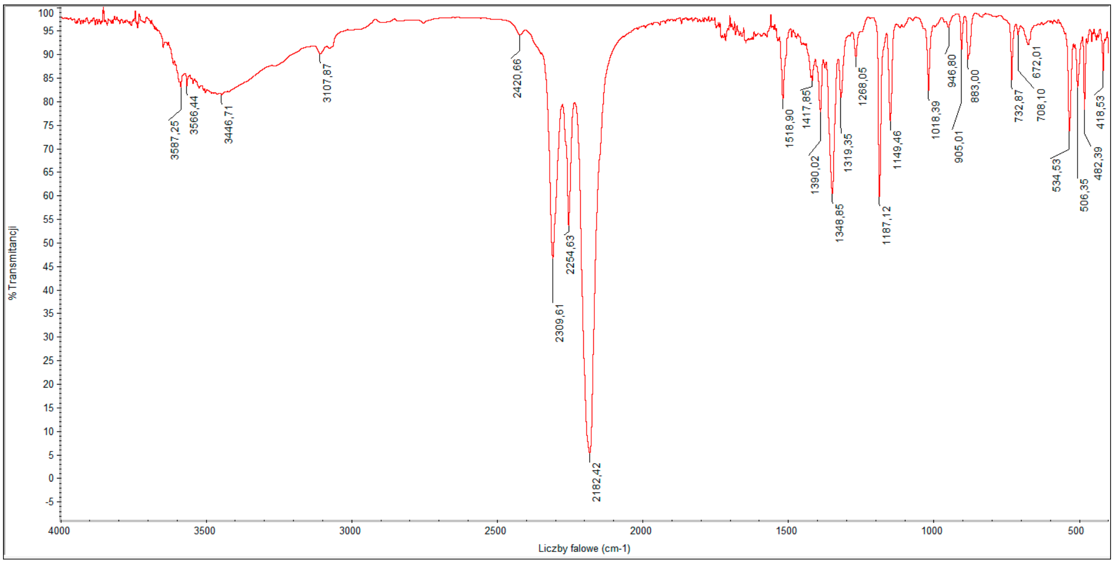
% Transmitancji
Liczby falowe (cm-1)
100
95
90
85
80
75
70
65
60
55
50
45
40
35
30
25
20
15
10
5
0
-5
4000
3500
3000
2500
2000
1500
1000
500
3587,25
3566,44
3446,71
3107,87
2420,66
2309,61
2254,63
2182,42
1518,90
1417,85
1390,02
1348,85
1319,35
1268,05
1187,12
1149,46
1018,39
946,80
905,01
883,00
732,87
708,10
672,01
534,53
506,35
482,39
418,53

6

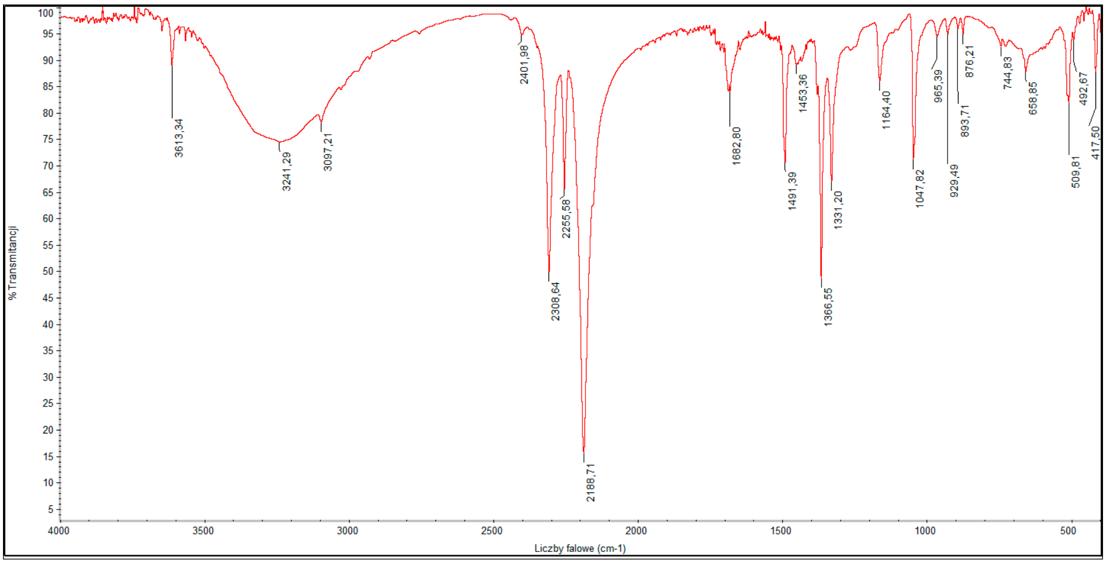
% Transmitancji
Liczby falowe (cm-1)
100
95
90
85
80
75
70
65
60
55
50
45
40
35
30
25
20
15
10
5
4000
3500
3000
2500
2000
1500
1000
500
3613,34
3241,29
3097,21
2401,98
2308,64
2255,58
2188,71
1682,80
1491,39
1453,36
1366,55
1331,20
1164,40
1047,82
965,39
929,49
893,71
876,21
744,83
658,85
509,81
492,67
417,50

7

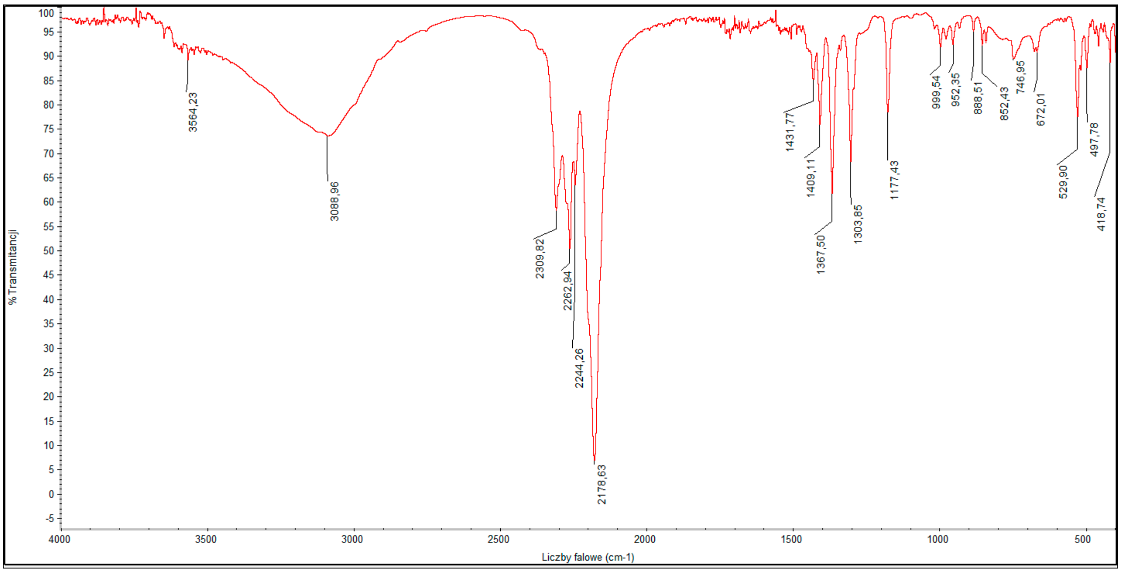
% Transmitancji
Liczby falowe (cm-1)
3564,23
3088,96
2309,82
2262,94
2244,26
2178,63
1431,77
1409,11
1367,50
1303,85
1177,43
999,54
952,35
888,51
852,43
746,95
672,01
529,90
497,78
418,74

8

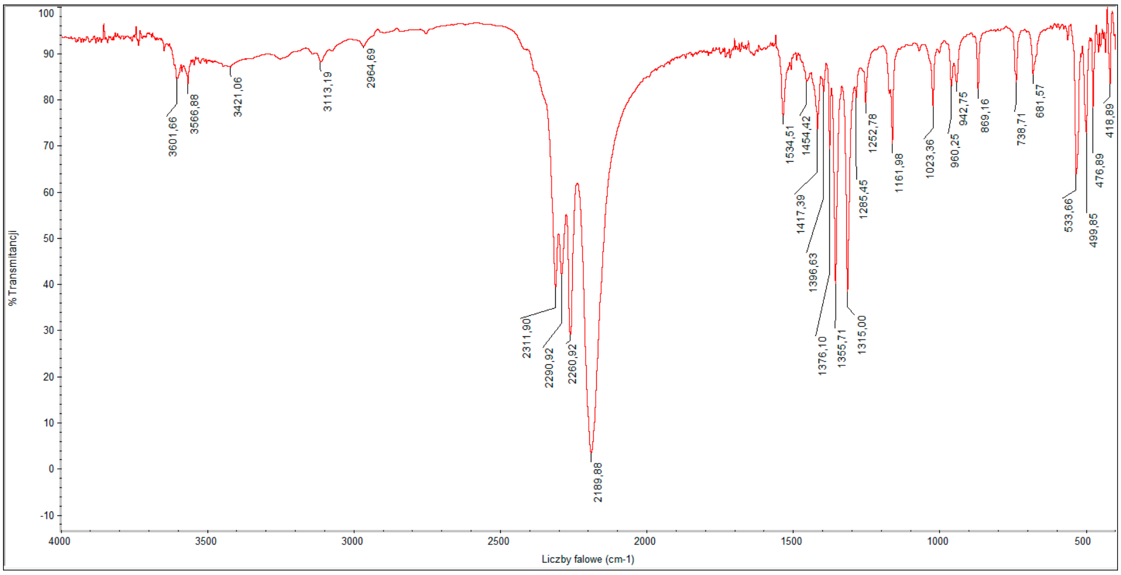
% Transmitancji
Liczby falowe (cm-1)
3601,66
3566,88
3421,06
3113,19
2964,69
2311,90
2290,92
2260,92
2189,88
1534,51
1454,42
1417,39
1396,63
1376,10
1355,71
1315,00
1285,45
1252,78
1161,98
1023,36
960,25
942,75
869,16
738,71
681,57
533,66
499,85
476,89
418,89

9

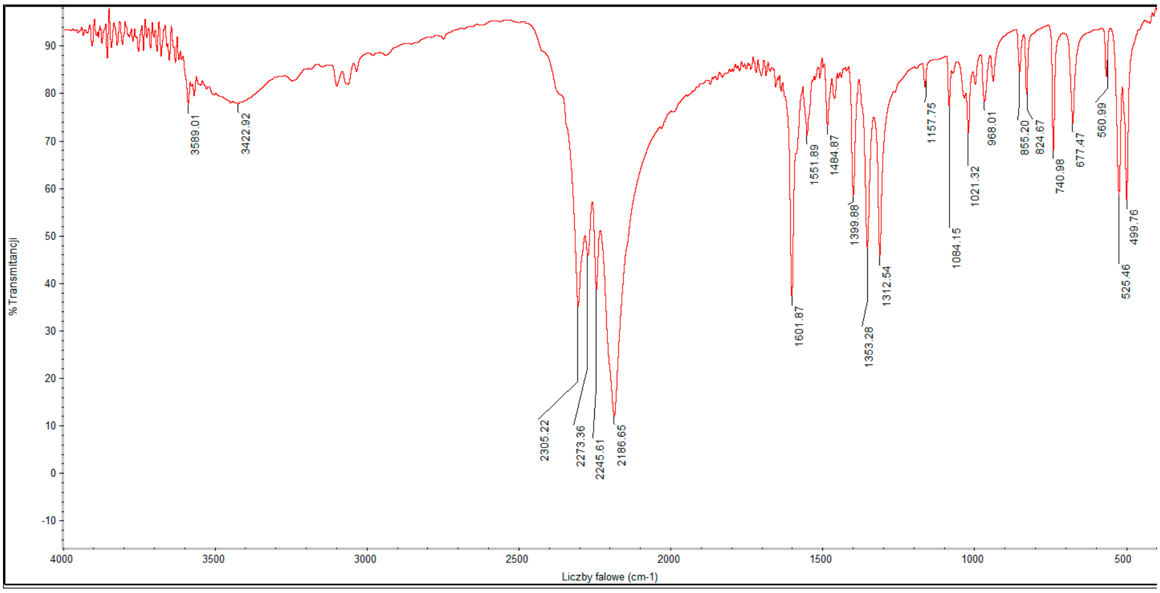
% Transmitancji
Liczby falowe (cm-1)
3589.01
3422.92
2305.22
2273.36
2245.61
2186.65
1601.87
1551.89
1484.87
1399.88
1353.28
1312.54
1157.75
1084.15
1021.32
968.01
855.20
824.67
740.98
677.47
560.99
525.46
499.76

10

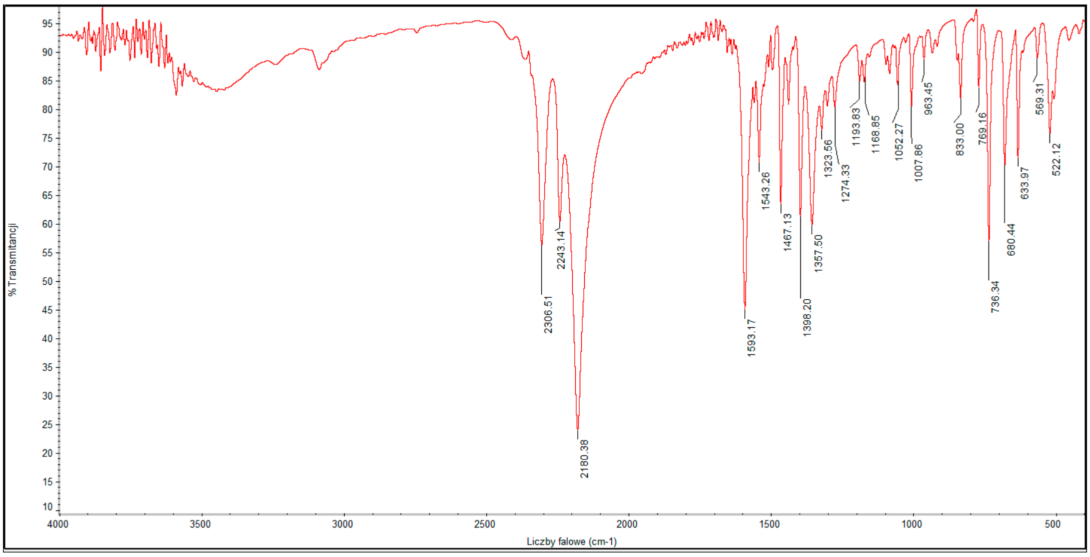
% Transmitancji
Liczby falowe (cm-1)
2306.51
2243.14
2180.38
1593.17
1543.26
1467.13
1398.20
1357.50
1323.56
1274.33
1193.83
1168.85
1052.27
1007.86
963.45
833.00
769.16
736.34
680.44
633.97
569.31
522.12

11

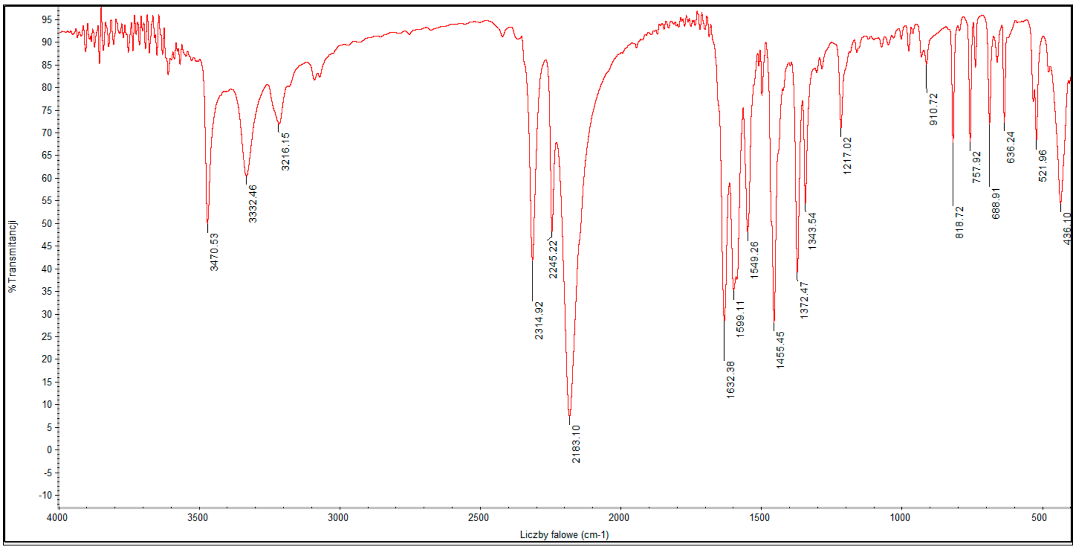
% Transmitancji
Liczby falowe (cm-1)
3470.53
3332.46
3216.15
2314.92
2245.22
2183.10
1632.38
1599.11
1549.26
1455.45
1372.47
1343.54
1217.02
910.72
818.72
757.92
688.91
636.24
521.96
436.10

12

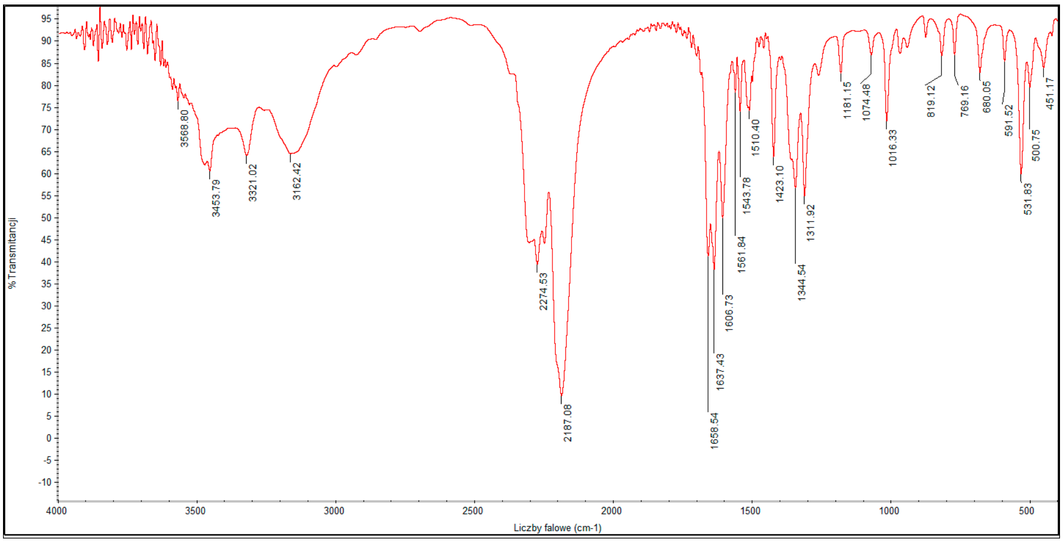
% Transmitancji
Liczby falowe (cm-1)
3568.80
3453.79
3321.02
3162.42
2274.53
2187.08
1658.54
1637.43
1606.73
1561.84
1543.78
1510.40
1423.10
1344.54
1311.92
1181.15
1074.48
1016.33
819.12
769.16
680.05
591.52
531.83
500.75
451.17

13

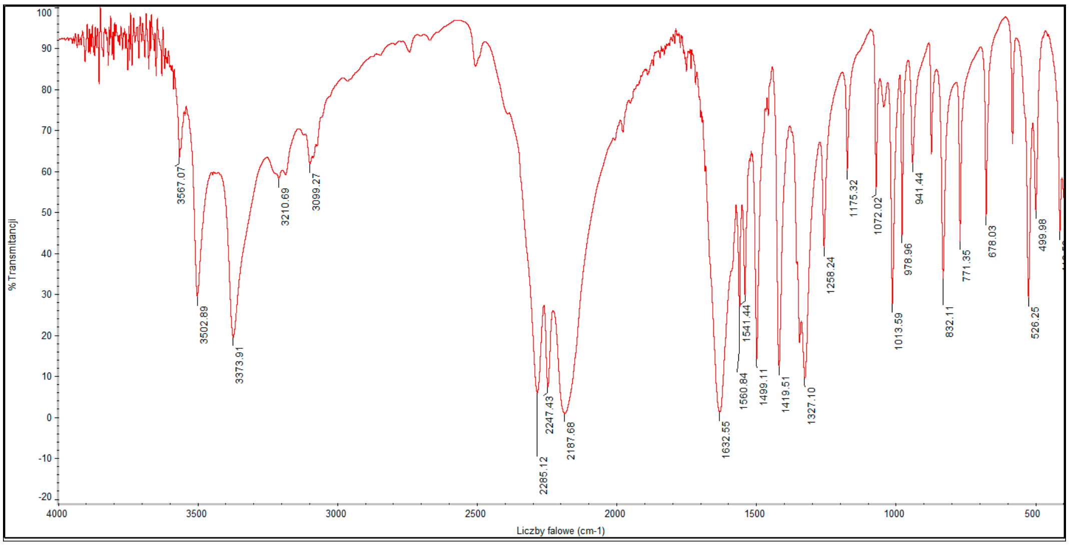

Figure S1. IR spectra of **1-13**.

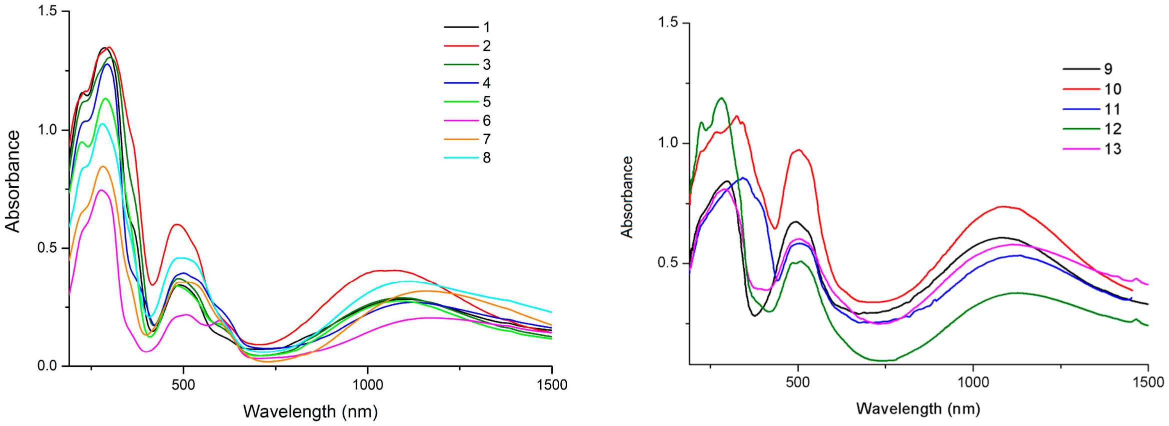

a **b**

Figure S2. UV-Vis spectra of powder samples of heteroleptic cobalt(II) dicyanamide systems with pyrazines (**a**) and pyrimidines (**b**) co-ligands.

1

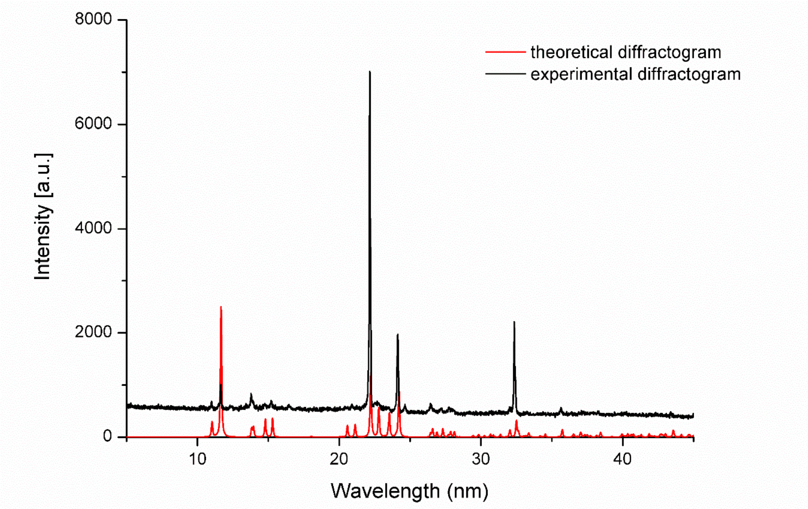
theoretical diffractogram
experimental diffractogram
Intensity [a.u.]
Wavelength (nm)

2

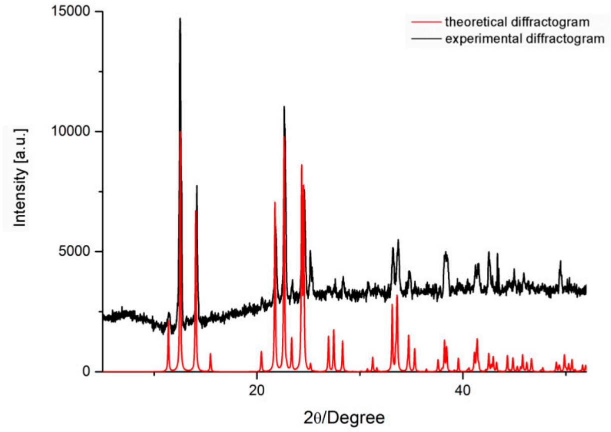
theoretical diffractogram
experimental diffractogram
Intensity [a.u.]
2θ/Degree

3

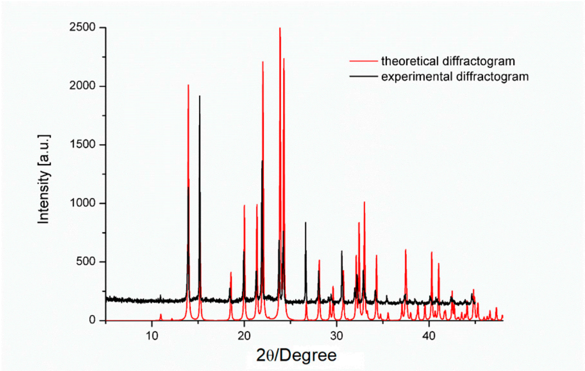
theoretical diffractogram
experimental diffractogram
Intensity [a.u.]
2θ/Degree

4

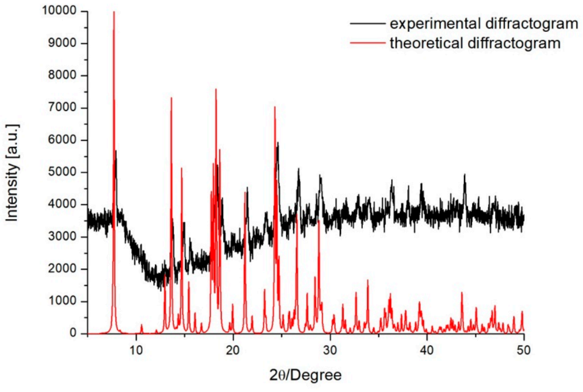
experimental diffractogram
theoretical diffractogram
Intensity [a.u.]
2θ/Degree

5

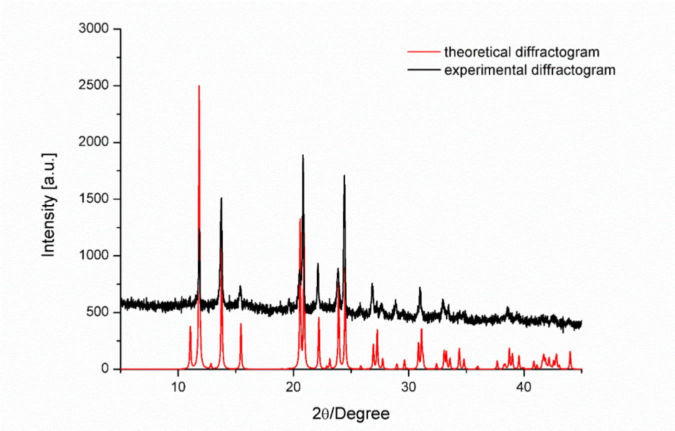
theoretical diffractogram
experimental diffractogram
Intensity [a.u.]
2θ/Degree
3000
2500
2000
1500
1000
500
0
10
20
30
40

6

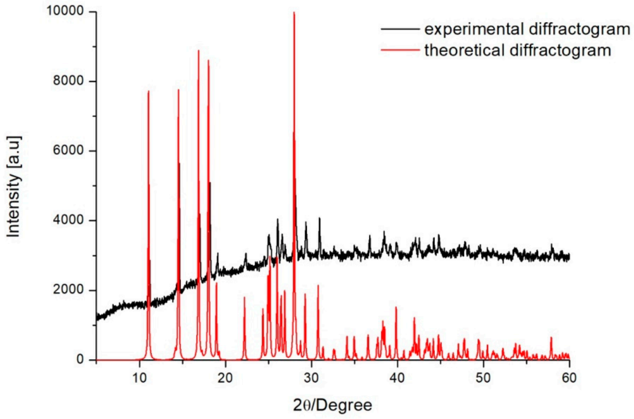
experimental diffractogram
theoretical diffractogram
Intensity [a.u]
2θ/Degree
10000
8000
6000
4000
2000
0
10
20
30
40
50
60

7

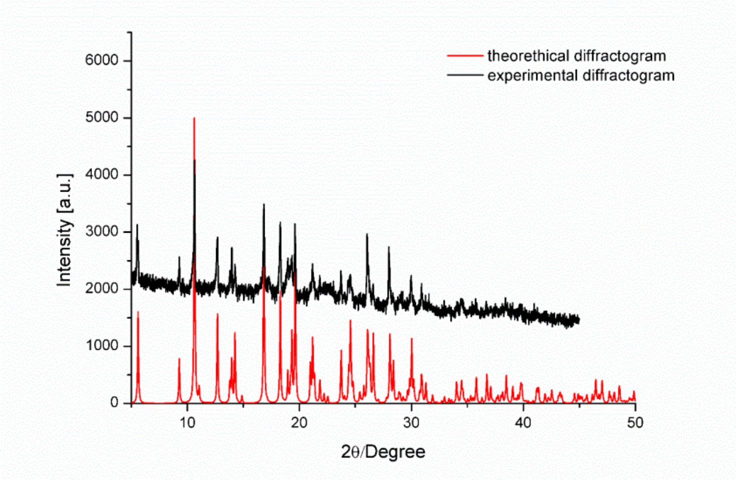
theorethical diffractogram
experimental diffractogram
Intensity [a.u.]
2θ/Degree
6000
5000
4000
3000
2000
1000
0
10
20
30
40
50

8

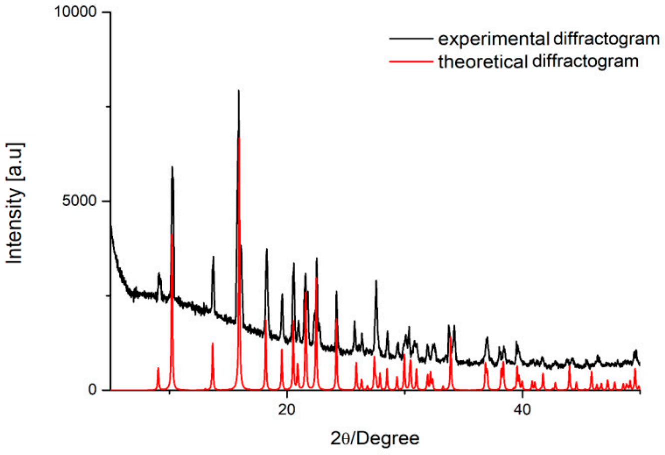
10000
5000
0
20
40
Intensity [a.u]
2θ/Degree
experimental diffractogram
theoretical diffractogram

9

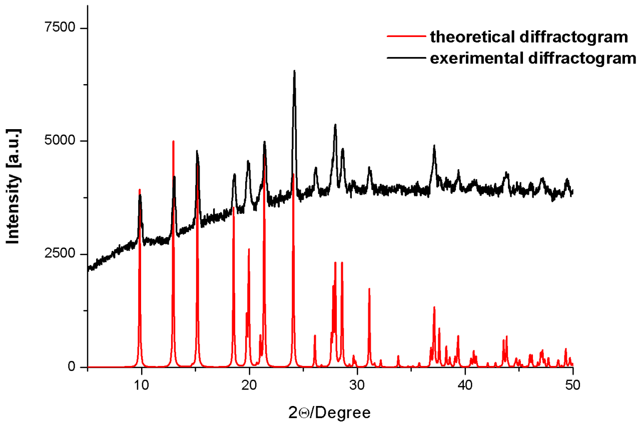
7500
5000
2500
0
10
20
30
40
50
Intensity [a.u.]
2Θ/Degree
theoretical diffractogram
exerimental diffractogram

10

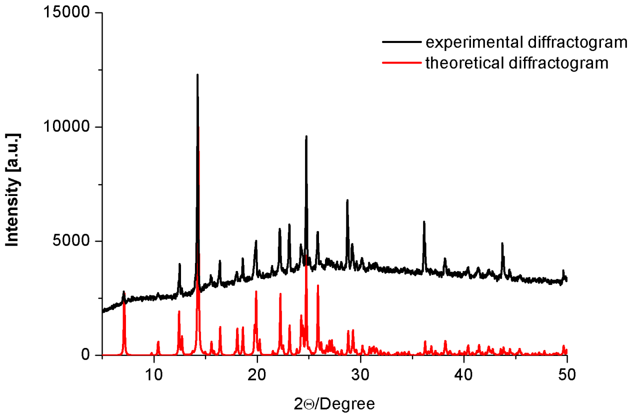
15000
10000
5000
0
10
20
30
40
50
Intensity [a.u.]
2Θ/Degree
experimental diffractogram
theoretical diffractogram

11

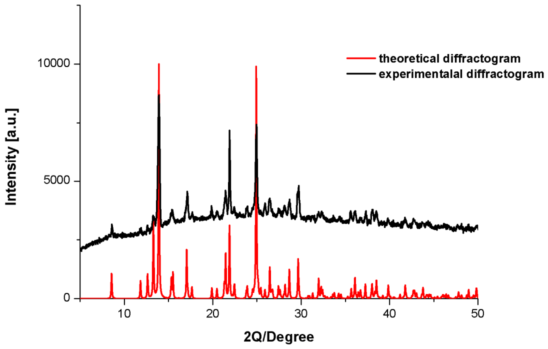

12

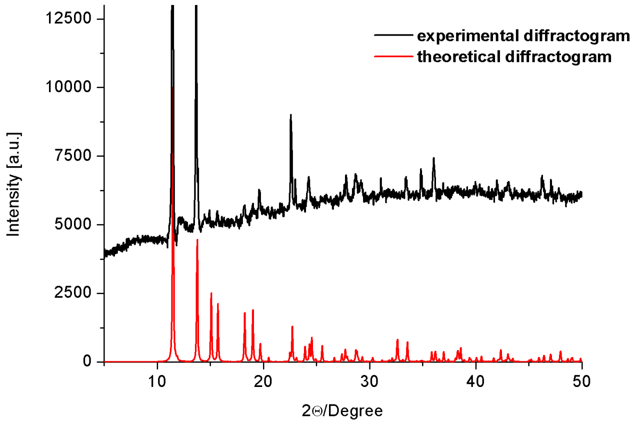

13

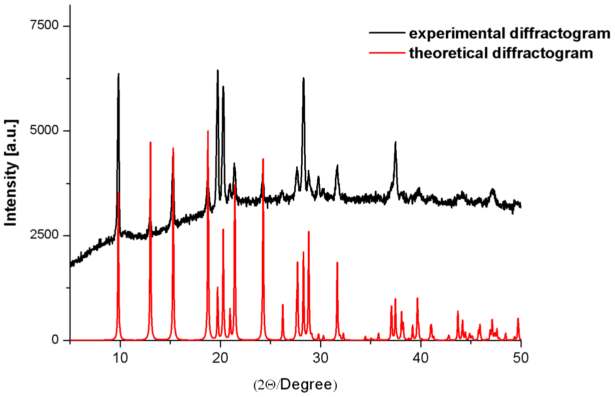

Figure S3. The powder XRPD pattern of **1-13** (experimental - black) and the simulation of its powder pattern from the crystal structure (red).

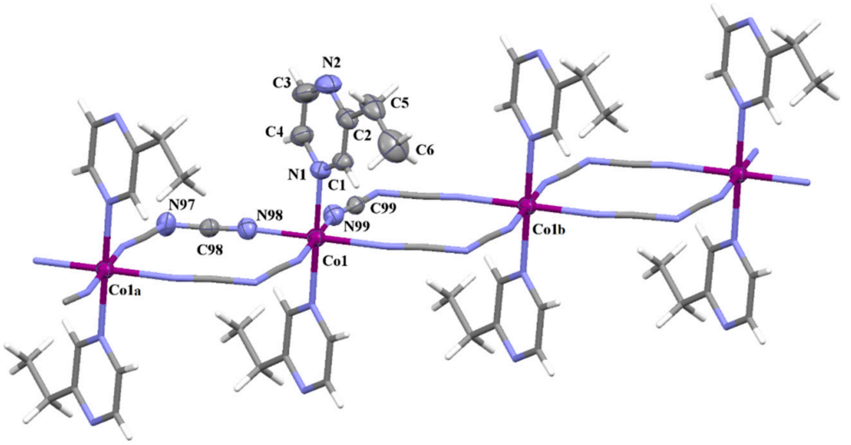

a

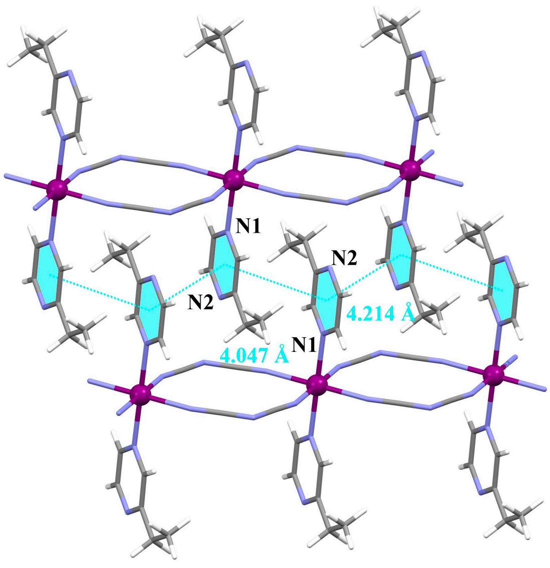

b

Figure S4. Coordination chain of **1** shown along the crystallographic *a* axis. Displacement ellipsoids are drawn at 50% probability level [symmetry codes: (a) = 1+*x*, *y*, *z*; (b) = –1+*x*, *y*, *z*]; b) A view of 2D supramolecular network of 1 generated through π–π type interactions.

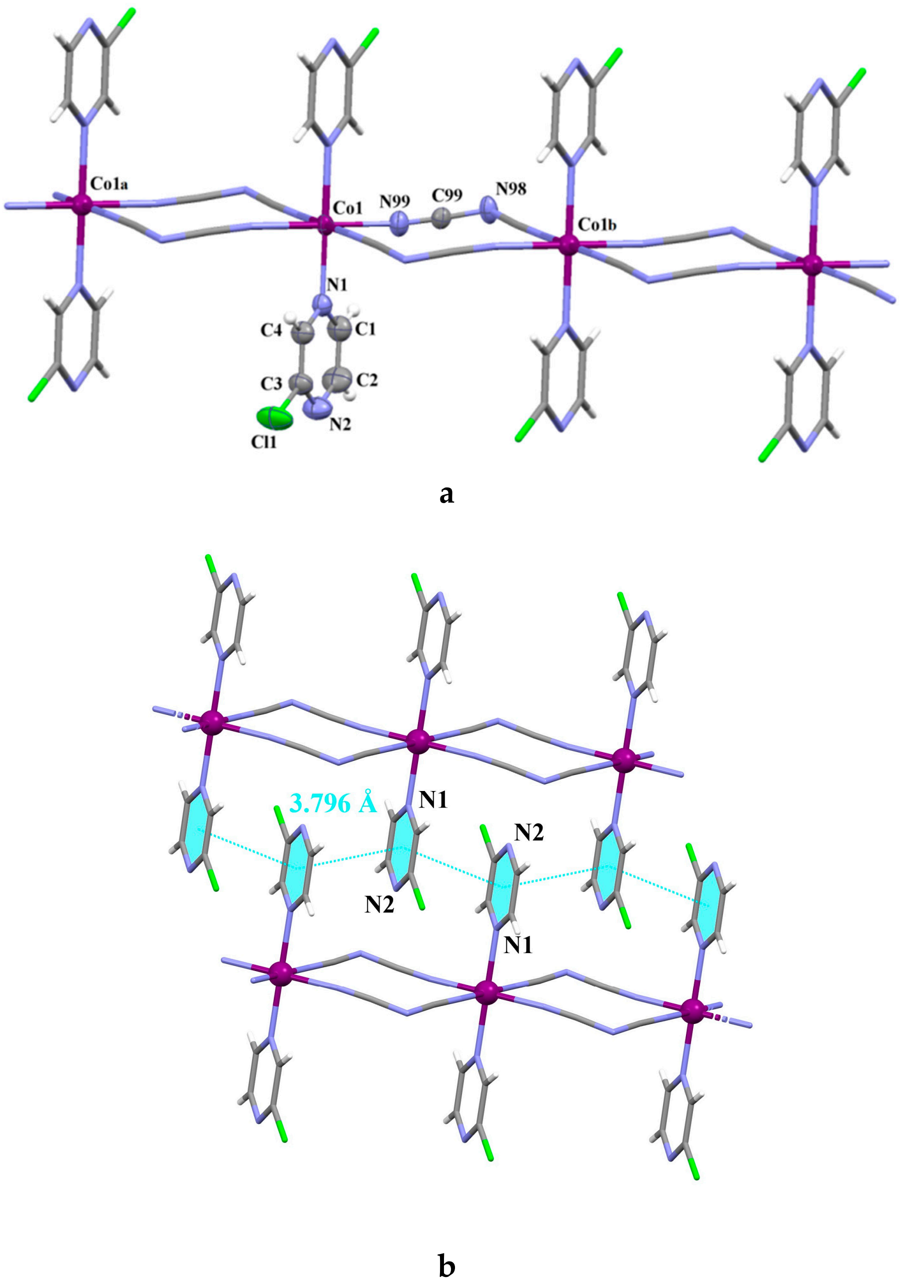

Figure S5. a) One-dimensional coordination network of **2** shown along the crystallographic *b* axis. Displacement ellipsoids are drawn at 50% probability level. b) View of a fragment of the 2D supramolecular structure of 2 generated through π–π type interactions.

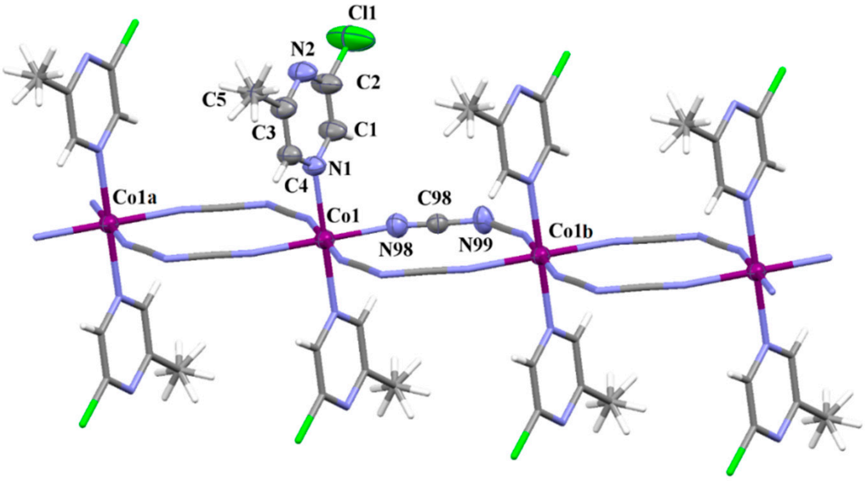

a

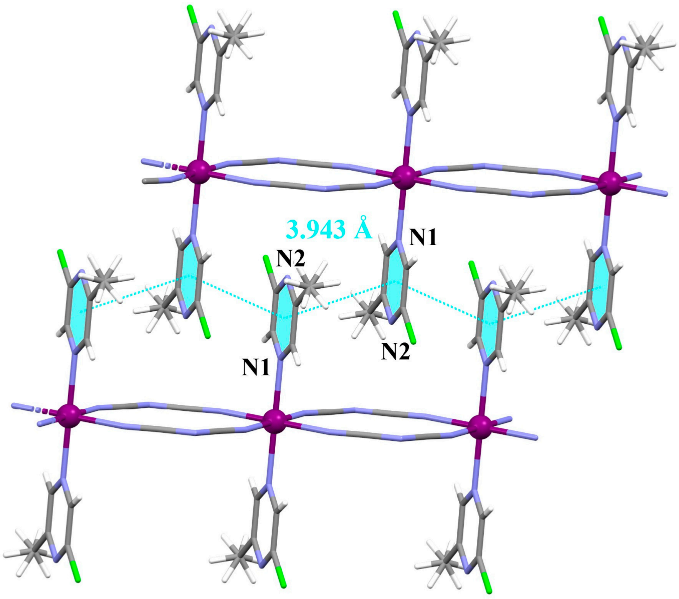

b

Figure S6. One-dimensional coordination network of **5** shown along the crystallographic *b* axis. Displacement ellipsoids are drawn at 50% probability level. [symmetry code: (a) = x, −1+*y*, *z*; (c) = *x*, 1+*y*, *z*]. (b) A view of 2D supramolecular structure of 5 generated through π–π type interactions.

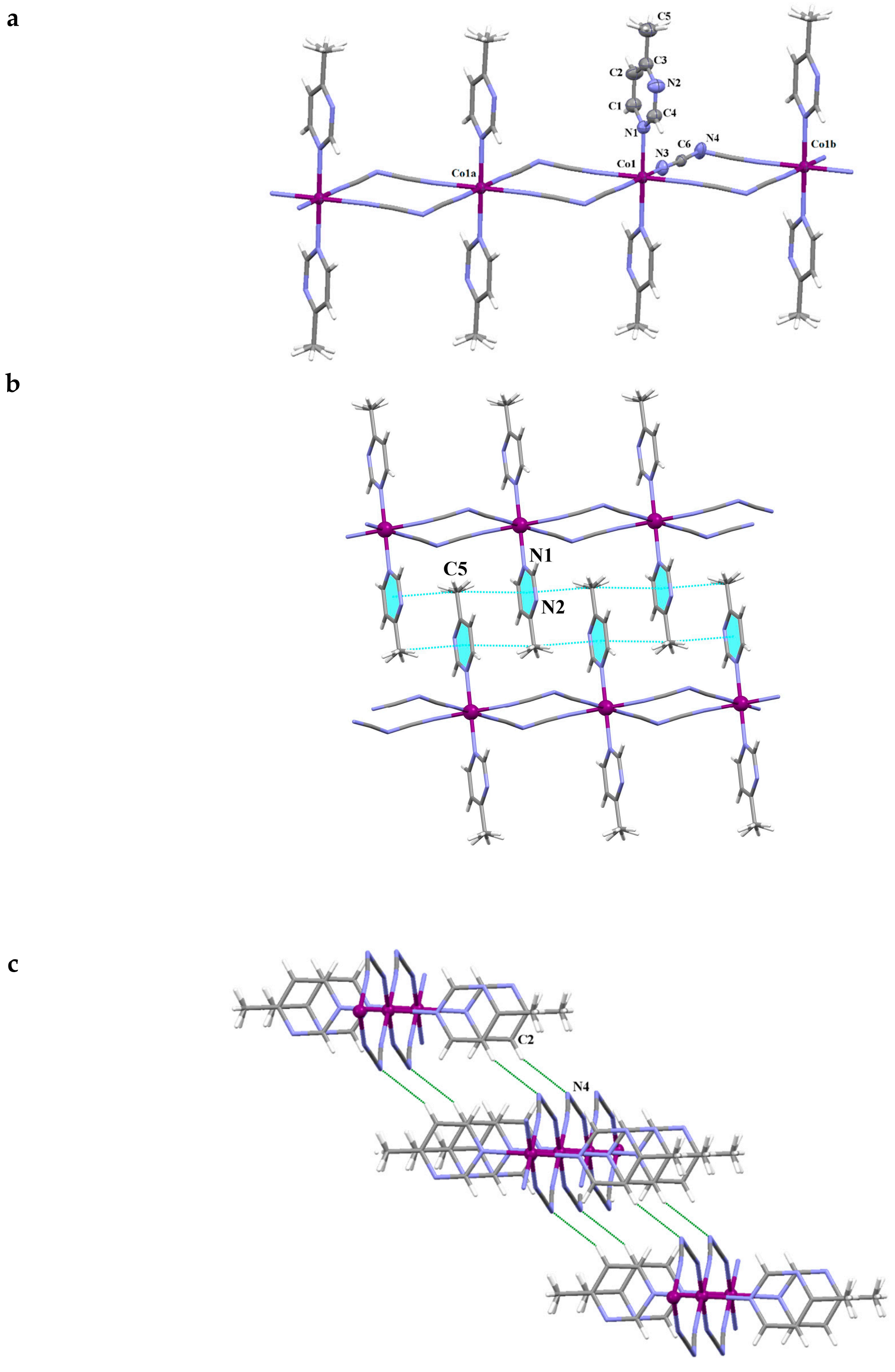

Figure S7. (a) Coordination chain of **9** shown along the crystallographic *b* axis. Displacement ellipsoids are drawn at 50% probability level [symmetry codes: (a) = *x*, 1+*y*, *z*; (b) = *x*, –1+*y*, *z*]; b) A view of crystal packing of **9** showing C–H•••π type interactions; c) Crystal packing of 9 showing the C–H•••N type short contacts.

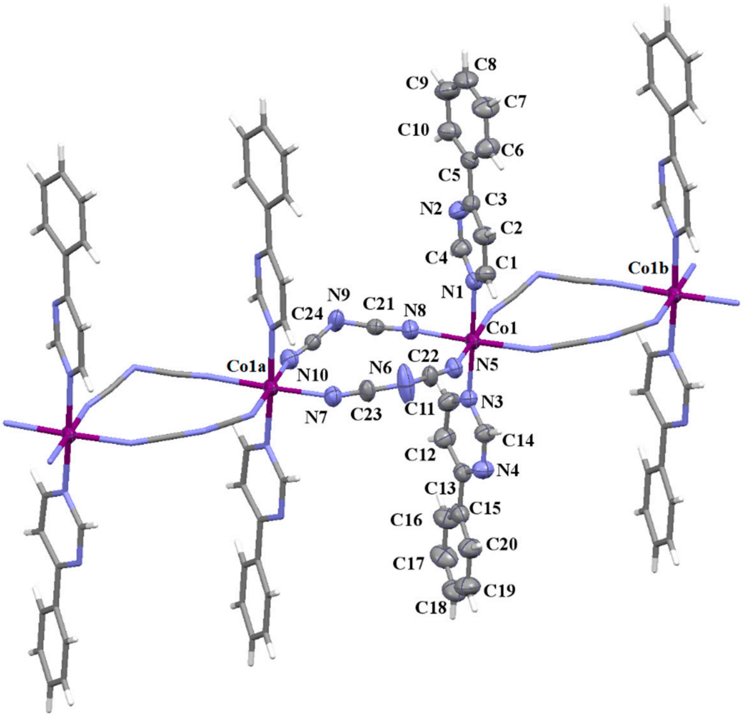

a

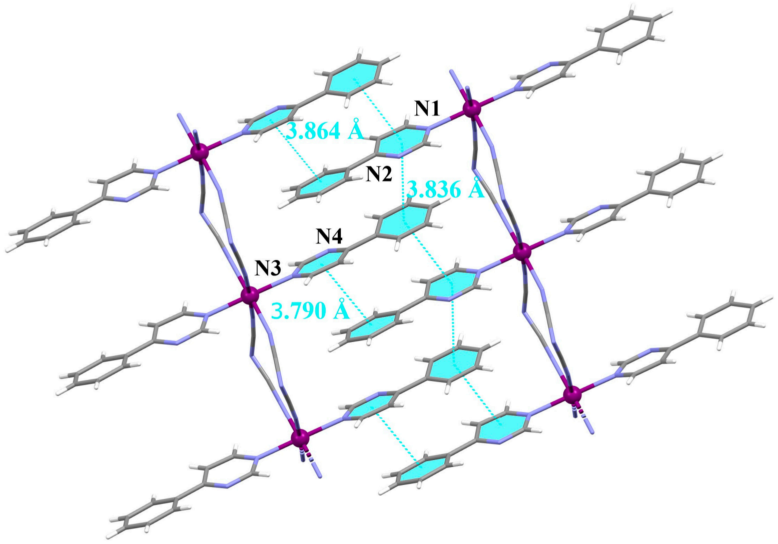

b

Figure S8. (a) Coordination chain of **10** shown along the crystallographic *a* axis. Displacement ellipsoids are drawn at 50% probability level [symmetry codes: (a) = 1+*x*, *y*, *z*; (b) = –1+*x*, *y*, *z*]; b) Crystal packing of **10** showing π •••π type interactions.

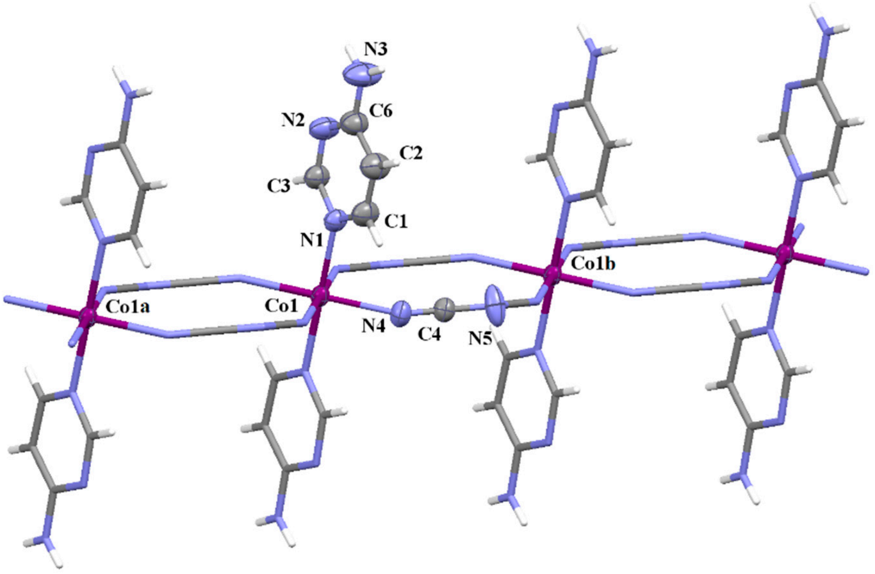

(a)

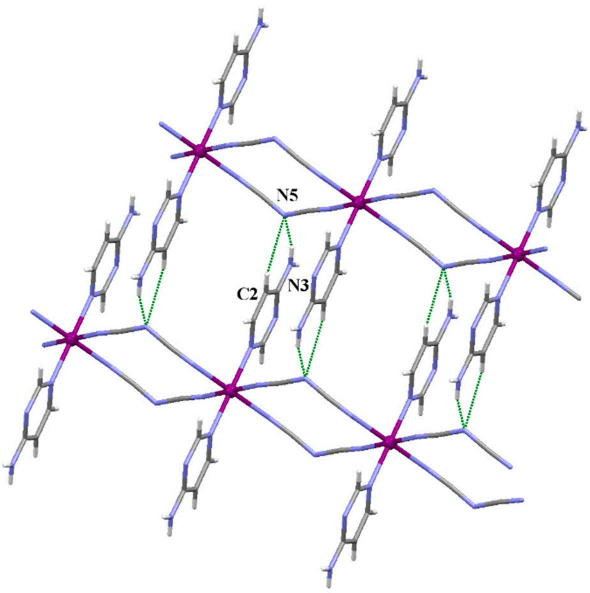

(b)

Figure S9. (a) Coordination chain of **12** shown along the crystallographic *b* axis. Displacement ellipsoids are drawn at 50% probability level [symmetry codes: (a) = *x*, –1+*y*, *z*; (b) = *x*, 1+*y*, *z*]; (b) The view of fragment of two-dimensional coordination network of 12 formed by N–H•••N and C–H•••N contacts.

**1** Co(1)<br>[Co(dca)$_2$(2–Etpyz)$_2$]$_n$	**2** Co(1)<br>[Co(dca)$_2$(2–Clpyz)$_2$]$_n$	**3** Co(1)<br>[Co(dca)$_2$(2–Ipyz)$_2$]$_n$
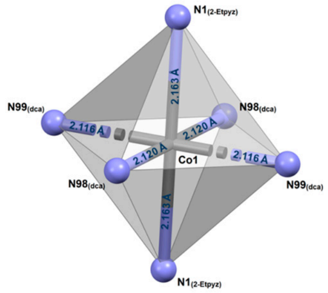	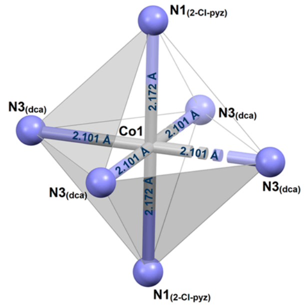	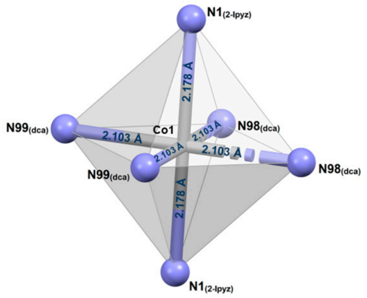
S(OC-6) = 0.026	S(OC-6) = 0.040	S(OC-6) = 0.054
**4** Co(1)<br>[Co$_3$(dca)$_6$(2–MeOpyz)$_4$]$_n$	**4** Co(2)<br>[Co$_3$(dca)$_6$(2–MeOpyz)$_4$]$_n$	**5** Co(1)<br>[Co(dca)$_2$(2–Cl–6–Mepyz)$_2$]$_n$
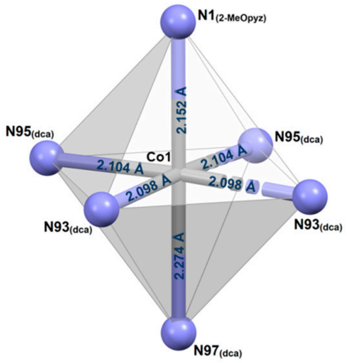	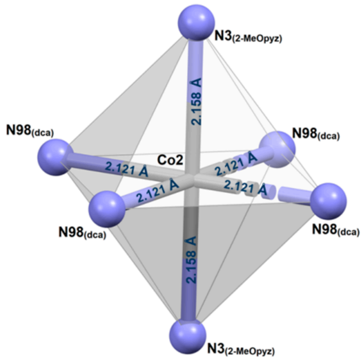	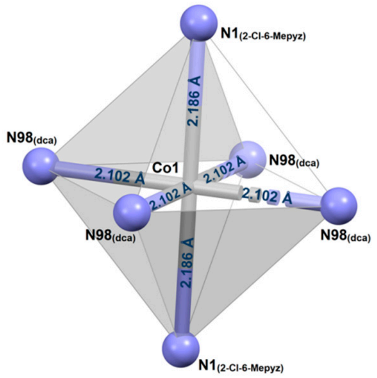
S(OC-6) = 0.109	S(OC-6) = 0.027	S(OC-6) = 0.036
**6** Co(1)<br>{[Co(dca)$_2$(H$_2$O)$_2$]·(2,5–Me$_2$pyz)}$_n$	**7** Co(1)<br>{[Co$_3$(dca)$_6$(2,3–Me$_2$pyz)$_2$(H$_2$O)$_2$]·2(2,3–Me$_2$pyz)}$_n$	**7** Co(2)<br>{[Co$_3$(dca)$_6$(2,3–Me$_2$pyz)$_2$(H$_2$O)$_2$]·2(2,3–Me$_2$pyz)}$_n$
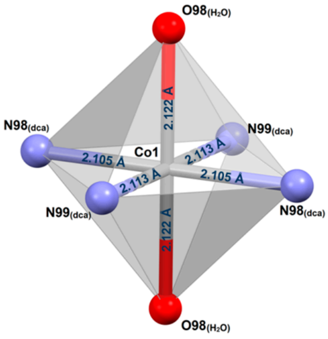	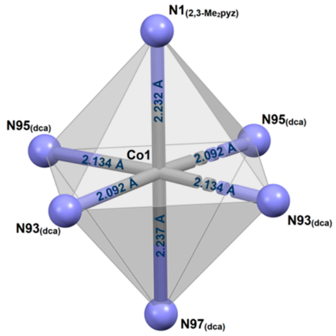	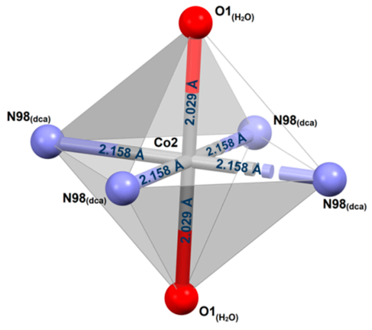
S(OC-6) = 0.026	S(OC-6) = 0.221	S(OC-6) = 0.247
**8** Co(1)<br>[Co(dca)$_2$(2,6–Me$_2$pyz)]$_n$	**8** Co(2)<br>[Co(dca)$_2$(2,6–Me$_2$pyz)]$_n$	**9**<br>[Co(dca)$_2$(4-Mepym)$_2$]$_n$

<table>
<tr><td>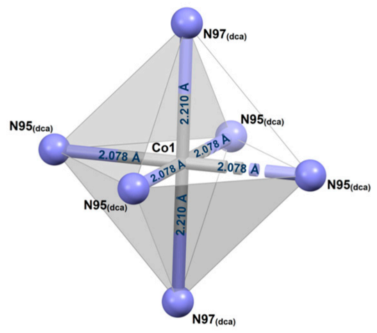
</td><td>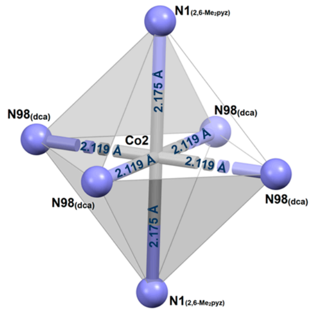
</td><td>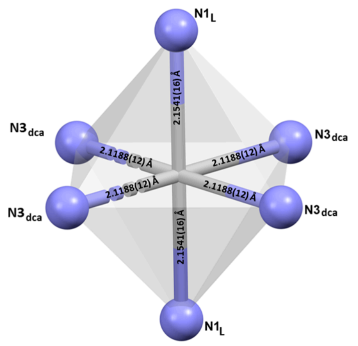
</td></tr>
<tr><td>S(OC-6) = 0.147</td><td>S(OC-6) = 0.039</td><td>S(OC-6) = 0.023</td></tr>
<tr><td>10
Co(dca)2(4-Phpym)2]n</td><td>11
[Co(dca)2(2-NH2-4-Phpym)2]n</td><td>12
[Co(dca)2(4-NH2pym)2]n</td></tr>
<tr><td>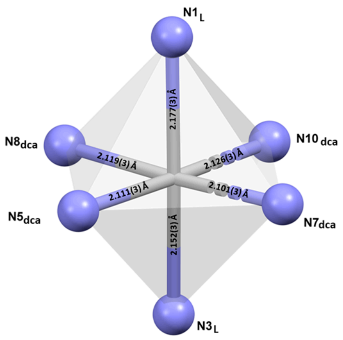
</td><td>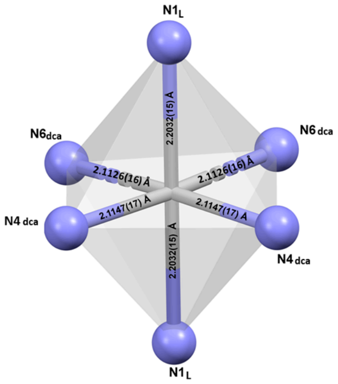
</td><td>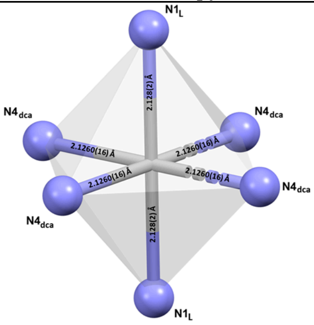
</td></tr>
<tr><td>S(OC-6) = 0.045</td><td>S(OC-6) = 0.102</td><td>S(OC-6) = 0.015</td></tr>
<tr><td colspan="3">13
[Co(dca)2(4-NH2pym)]n</td></tr>
<tr><td colspan="3">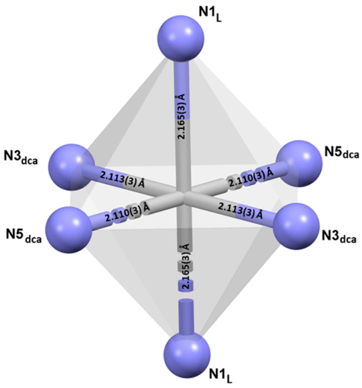
</td></tr>
<tr><td colspan="3">S(OC-6) = 0.103</td></tr>
</table>

Figure S10. The cobalt environment in **1–13** together with shape values (SQ(P)) with respect to the octahedral geometry (OC-6), calculated with SHAPE program. The shape measure $S_Q(P)$ is defined as $S_Q(P)=\min\left[\left(\sum_{i=1}^{n}|\vec{q}_i-\vec{p}_i|^2\right)/\left(\sum_{i=1}^{n}|\vec{q}_i-\vec{q}_0|^2\right)\right]\times 100$, where $\overrightarrow{q_i}$ are N vectors that contain the $3N$ Cartesian coordinates of the problem structure Q; $\overrightarrow{p_i}$ contain the coordinates of the ideal polyhedron P; $\overrightarrow{q_0}$ is the position vector of the geometric centre that is chosen to be the same for the two polyhedra.

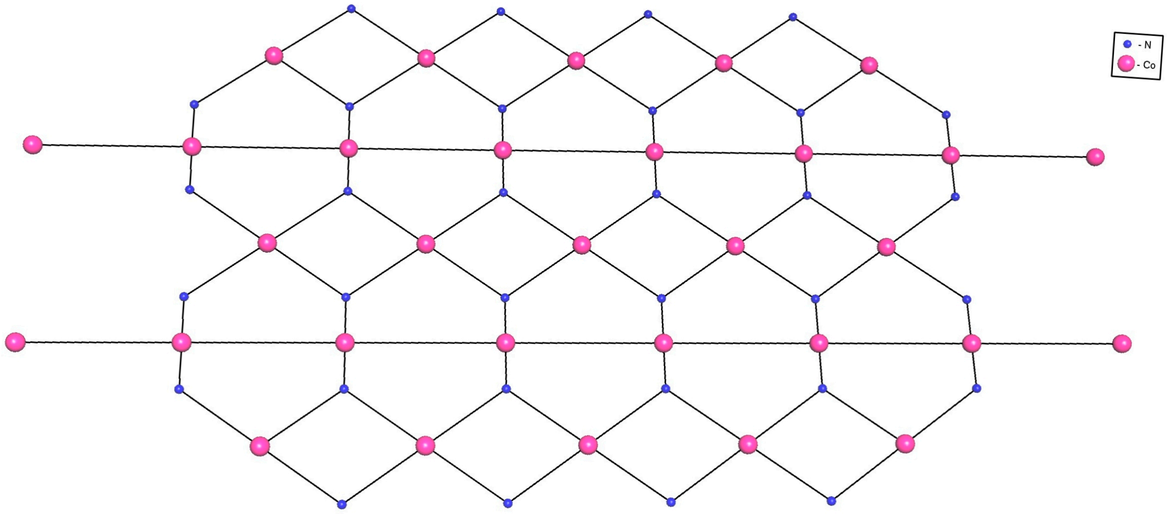

Figure S11. Coordination polymer framework of **8**.

Table S3. Selected bond lengths (Å) and angles (deg) for **1–8.**

	**1**	**2**	**3**	**4**	**5**	**6**	**7**	**8**
**Bond distances**								
Co(1)–O(98)						2.122(2)		
Co(1)–N(1)	2.163(3)	2.172(2)	2.176(5)	2.152(3)	2.186(5)		2.232(3)	
Co(2)–N(1)								2.175(4)
Co(1)–N(93)				2.093(2)			2.134(3)	
Co(1)–N(95)				2.104(2)			2.092(3)	2.078(4)
Co(1)–N(97)				2.274(2)			2.237(3)	2.210(4)
Co(1)–N(98)	2.120(3)		2.105(4)		2.102(4)	2.105(2)		
Co(1)–N(99)	2.116(3)	2.1007(16)				2.113(3)		
Co(2)–N(3)				2.158(2)				
Co(2)–N(98)				2.121(2)			2.158(3)	2.119(3)
Co(2)–O(1)							2.029(3)	
N(93)–C(94)				1.151(3)			1.145(4)	
N(94)–C(94)				1.303(3)			1.302(4)	
N(95)–C(96)				1.152(3)			1.148(4)	1.141(6)
N(96)–C(96)				1.302(3)			1.303(4)	1.292(5)
N(97)–C(98)	1.298(5)		1.302(5)	1.321(2)		1.322(3)	1.319(4)	1.317(5)
N(97)–C(99)	1.304(5)					1.302(3)		
N(98)–C(98)	1.142(4)		1.147(6)	1.143(3)	1.146(6)	1.145(3)	1.155(4)	1.143(5)
N(98)–C(98)					1.291(5)			
N(99)–C(99)	1.151(4)							
N(99)–C(99n)						1.174(3)		
	**1**	**2**	**3**	**4**	**5**	**6**	**7**	**8**
**Bond angles**								
N(98)–Co(1)–O(98)						90.79(8)		
N(98)–Co(1)–O(98o)						89.21(8)		
N(99)–Co(1)–O(98)						90.15(9)		
N(99)–Co(1)–O(98o)						89.85(9)		
O(98)–Co(1)–O(98o)						180.0		
O(1)–Co(2)–O(1s)							180.0	
O(1)–Co(2)–N(98)							89.97(10)	
O(1)–Co(2)–N(98s)							90.03(10)	
N(1)–Co(1)–N(1)		180.0						
N(1)–Co(1)–N(1p)	180.0							

N(1)–Co(1)–N(1d)			180.0					
N(1)–Co(1)–N(1l)					180.0			
N(1)–Co(1)–N(93)				93.38(8)			89.22(9)	
N(1)–Co(1)–N(95)				89.84(8)			93.24(9)	
N(1)–Co(1)–N(97)				179.08(11)			179.42(13)	
N(1)–Co(1)–N(98)	89.38(11)		91.33(14)		90.32(15)			
N(1)–Co(1)–N(98d)			88.67(14)					
N(1)–Co(1)–N(98c)	90.62(11)							
N(1)–Co(1)–N(98l)					89.68(15)			
N(1)–Co(1)–N(99)	89.12(11)	91.11(6)						
N(1)–Co(1)–N(99c)	90.88(11)							
N(1)–Co(1)–N(99i)		88.89(6)						
N(93)–Co(1)–N(93h)				91.77(12)				
N(93)–Co(1)–N(93g)							92.82(15)	
N(93)–Co(1)–N(95)				88.52(8)			85.56(11)	
N(93)–Co(1)–N(95g)							177.04(10)	
N(93)^{h}–Co(1)–N(95)				176.74(8)				
N(93)–Co(1)–N(97)				87.26(7)			90.38(9)	
N(95)–Co(1)–N(95h)				91.01(12)				
N(95)–Co(1)–N(95g)							95.95(15)	
N(95)–Co(1)–N(95p)								180.0
N(95)–Co(1)–N(95u)								86.5(2)
N(95)–Co(1)–N(95v)								93.5(2)
N(95)–Co(1)–N(97)				89.52(7)			87.15(9)	90.0
N(97)–Co(1)–N(97p)								180.0
N(98)–Co(1)–N(98p)	180.0							
N(98)–Co(1)–N(98c)					180.0			
N(98)–Co(1)–N(98f)					90.0(2)			
N(98)–Co(1)–N(98d)			180.0					
N(98)–Co(1)–N(98l)					180.0			
N(98)–Co(1)–N(98o)						180.0		
N(98d)–Co(1)–N(98e)			91.2(2)					
N(98d)–Co(1)–N(98f)			88.8(2)					
N(98)–Co(1)–N(99)	91.36(12)					92.08(9)		
N(98)–Co(1)–N(99c)	88.64(12)							
N(98)–Co(1)–N(99o)						87.92(9)		
N(99)–Co(1)–N(99f)		90.66(9)						
N(99)–Co(1)–N(99i)		180.0						
N(99)–Co(1)–N(99p)	180.0							
N(99)–Co(1)–N(99aa)		89.34(9)						
N(99i)–Co(1)–N(99aa)		180.00(6)						
N(1)–Co(2)–N(1l)								180.0
N(1)–Co(2)–N(98l)								90.0
N(3)–Co(2)–N(3i)				180.0				
N(3)–Co(2)–N(98)				89.04(3)				
N(3)–Co(2)–N(98i)				90.96(6)				
N(98)–Co(2)–N(98j)				88.55(9)				
N(98)–Co(2)–N(98k)				91.45(9)				
N(98)–Co(2)–N(98i)				180.0				
N(98)–Co(2)–N(98f)							95.64(14)	
N(98)–Co(2)–N(98t)							84.36(14)	
N(98)^{f}–Co(2)–N(98t)							180.0	
N(98)–Co(2)–N(98l)								180.0
N(98)–Co(2)–N(98h)								87.8(2)
N(98)–Co(2)–N(98x)								92.2(2)
Co(1)–N(1)–C(1)	120.7(2)		118.2(4)	121.6(2)	120.3(4)		130.0(3)	
Co(1)–N(1)–C(4)	122.3(2)		125.2(4)	122.3(3)	122.4(4)		113.6(6)	
Co(1)–N(93)–C(94)				151.9(2)			158.4(3)	
Co(1)–N(95)–C(96)				159.8(2)			159.2(3)	160.6(4)
Co(1)–N(97)–C(98)				121.03(12)			120.62(17)	121.2(2)
Co(1)–N(98)–C(98)	162.0(3)		157.6(4)		160.8(4)	160.8(2)		
Co(1)–N(98)–C(98g)			118.4(5)					
Co(1)–N(99)–C(99)	159.1(3)	157.07(15)						
Co(1)–N(99)–C(99r)						163.6(2)		

Co(2)–N(1)–C(1)								121.7(2)
Co(2)–N(3)–C(6)				122.92(19)				
Co(2)–N(3)–C(9)				120.4(2)				
Co(2)–N(98)–C(98)				163.07(16)			165.2(2)	162.7(3)
N(93)–C(94)–N(94)				174.7(2)			173.4(4)	
N(95)–C(96)–N(96)				174.8(3)			174.2(3)	173.9(5)
N(98)–C(98)–N(97)	173.7(4)		174.4(5)	175.7(2)			175.8(3)	175.4(4)
N(98)–C(98)–N(97)								
N(99)–C(98)–N(99)	174.6(3)				175.3(5)			
N(99)–C(99)–N(98)		174.66(19)						
C(94)–N(94)–C(94f)							122.3(4)	
C(96)–N(96)–C(96f)							121.7(4)	
C(96)–N(96)–C(96q)				118.7(3)				
C(96)–N(96)–C(96y)								119.9(6)
C(98)–N(97)–C(98g)							116.6(3)	
C(98)–N(97)–C(98h)				117.0(2)				
C(98)–N(97)–C(98z)								117.6(5)
C(98)–N(97)–C(99a)	118.9(3)							
C(98)–N(99)–C(98m)					118.9(6)			
C(99)–N(98)–C(99m)		119.2(2)						

Symmetry codes: (a) = 1+*x*, *y*, *z*; (b) = –1+*x*, *y*, *z*; (c) = –*x*, *y*, 1–*z*; (d) = –*x*, –*y*, 1–*z*; (e) = –*x*, *y*, –*z*; (f) = *x*, 1–*y*, *z*; (g) = *x*, –*y*, *z*; (h) = *x*, *y*, 1–*z*; (i) = 1–*x*,1– *y*, 2–*z*; (j) = 1–*x*,1– *y*, *z*; (k) = *x*, *y*, 2–*z*; (l) = –*x*, 1–*y*, 1–*z*; (m) = *x*, 2–*y*, *z*; (n) = 1+*x*, 1+*y*, *z*; (o) = 1–*x*, –2–*y*, –1–*z*; (p) = –*x*, –*y*, –*z*; (q) = *x*, *y*, 2– *z*; (r) = –1+*x*,1+*y*, *z*; (s) = 1–*x*,1– *y*, 1–*z*; (t) = 1–*x*, *y*,1– *z*; (u) = –*x*, *y*, *z*; (v) = *x*, –*y*,– *z*; (x) = –*x*, 1–*y*, *z*; (y) = *x*, –1–*y*, –*z*; (z) = –*x*, –*y*, *z*; (aa) = 1–*x*, *y*, 2–*z*;

Table S4. Selected bond lengths (Å) and angles (deg) for **9–13.**

	**9**	**10**	**11**	**12**	**13**
**Bond lengths (Å)**					
Co(1)–N(1)	2.1541(16)	2.177(3)	2.2032(15)	2.128(2)	2.165(3)
Co(1)–N(3)	2.1188(12)	2.152(3)			2.113(3)
Co(1)–N(4)			2.1147(17)	2.1260(16)	
Co(1)–N(5)		2.111(3)			2.110(3)
Co(1)–N(6)			2.1126(16)		
Co(1)–N(7d)		2.101(3)			
Co(1)–N(8)		2.119(3)			
Co(1)–N(10)		2.126(3)			
N(3)–C(4)					1.128(5)
N(3)–C(6)	1.1461(18)				
N(4)–C(4)				1.140(2)	1.297(6)
N(4)–C(5)					1.298(6)
N(4)–C(6)	1.3023(16)				
N(4)–C(11)			1.143(3)		
N(4)–C(5i)					1.135(5)
N(5)–C(4)				1.305(2)	
N(5)–C(11)			1.287(3)		
N(5)–C(12)			1.284(3)		
N(5)–C(22)		1.129(4)			
N(6)–C(12f)			1.143(2)		
N(6)–C(22)		1.306(5)			
N(6)–C(23)		1.294(5)			
N(7)–C(23)		1.134(4)			
N(8)–C(21)		1.143(4)			
N(9)–C(21)		1.300(4)			
N(9)–C(24e)		1.295(4)			
N(10)–C(24)		1.146(4)			
**Bond angels (°)**					
N(1)–Co(1)–(N1a)	180.0			180.0	
N(1)–Co(1)–(N1j)					180.0
N(1)–Co(1)–(N1g)			179.14(8)		
N(1)–Co(1)–(N3)	89.57(5)	176.70(10)			89.86(13)
N(1)–Co(1)–(N3a)	90.43(5)				

N(1)–Co(1)–(N3j)					90.14(13)
N(1)–Co(1)–(N4)			89.15(6)	88.81(6)	
N(1)–Co(1)–(N4a)				91.19(6)	
N(1)–Co(1)–(N4g)			90.23(6)		
N(1)–Co(1)–(N5)		90.18(11)			90.03(13)
N(1)–Co(1)–(N5j)					89.97(13)
N(1)–Co(1)–(N6)			92.36(6)		
N(1)–Co(1)–(N6g)			88.26(6)		
N(1)–Co(1)–(N7d)		90.19(11)			
N(1)–Co(1)–N(8)		87.43(11)			
N(1)–Co(1)–N(10)		88.70(11)			
N(3)–Co(1)–(N3a)	180.0				
N(3)–Co(1)–(N3b)	91.71(7)				
N(3)–Co(1)–(N3j)					180.0
N(3a)–Co(1)–(N3b)	88.29(7)				
N(3)–Co(1)–(N5)		91.63(11)			89.68(14)
N(3)–Co(1)–(N5j)					90.32(14)
N(3)–Co(1)–(N7d)		92.51(11)			
N(3)–Co(1)–N(8)		89.89(10)			
N(3)–Co(1)–N(10)		89.47(11)			
N(4)–Co(1)–(N4a)				180.0	
N(4)–Co(1)–(N4c)				91.18(9)	
N(4)–Co(1)–(N4l)				88.82(9)	
N(4c)–Co(1)–(N4l)				180.00(9)	
N(4)–Co(1)–(N4g)			87.95(9)		
N(4)–Co(1)–(N6)			92.16(7)		
N(4)–Co(1)–(N6g)			177.41(6)		
N(5)–Co(1)–(N5j)					180.0
N(5)–Co(1)–(N7d)		91.78(11)			
N(5)–Co(1)–(N8)		88.46(11)			
N(5)–Co(1)–(N10)		178.84(11)			
N(6)–Co(1)–(N6g)			87.85(9)		
N(7d)–Co(1)–N(8)		177.61(11)			
N(7d)–Co(1)–N(10)		88.53(11)			
N(8)–Co(1)–N(10)		91.18(11)			
Co(1)–N(3)–C(4)					161.2(4)
Co(1)–N(3)–C(6)	157.59(12)				
Co(1)–N(4)–C(4)				156.57(16)	
Co(1)–N(4)–C(11)			157.36(16)		
Co(1)–N(5)–C(22)		152.5(3)			
Co(1)–N(5)–C(5k)					162.3(4)
Co(1)–N(6)–C(12f)			156.96(17)		
Co(1e)–N(7)–C(23)		159.5(3)			
Co(1)–N(8)–C(21)		162.5(3)			
Co(1)–N(10)–C(24)		152.0(3)			
C(4)–N(4)–C(4b)				118.0(2)	
C(4)–N(4)–C(5)					122.5(4)
C(6)–N(4)–(C6c)	119.93(17)				
C(11)–N(5)–(C12)			124.0(2)		
C(22)–N(6)–C(23)		121.0(3)			
C(21)–N(9)–C(24e)		120.5(3)			
N(3)–C(4)–N(4)					172.6(5)
N(3)–C(6)–N(4)	174.21(15)				
N(4)–C(4)–N(5)				174.7(2)	
N(4)–C(5)–N(5j)					172.1(5)
N(4)–C(11)–N(5)			173.5(2)		
N(5)–C(12)–N(6h)			173.6(2)		
N(5)–C(22)–N(6)		172.8(4)			
N(7)–C(23)–N(6)		173.1(4)			
N(8)–C(21)–N(9)		174.0(3)			
N(10)–C(24)–N(9d)		174.1(3)			

Symmetry codes: (a) = 1–*x*, 1–*y*, 1–*z*; (b) = *x*, 1– *y*, *z*; (c) = *x*, – *y*, *z*; (d) =–1+*x*, *y*, *z*; (e) =1+*x*, *y*, *z*; (f) = *x*, 1+*y*, *z*; (g) = –*x*, *y*, 1/2–*z*; (h) = *x*, –1+*y*, *z*; (i) = 1/2–*x*, –*y*, 1/2+*z*; (j) = –*x*, –*y*, –*z*; (k) = 1/2–*x*, –*y*, –1/2+*z*; (l) = 1–*x*, *y*, 1–*z*;

Table S5. Short π•••π interactions for **1-3**, **5**, **8**,**10** and **11**[a]

Cg(I)•••Cg(J)	Cg(I)•••Cg(J) [Å]	α[°][b]	β[°][c]	γ [°][d]	Cg(I)-Perp [Å][e]	Cg(J)-Perp [Å][f]
			**1**			
Cg(1)•••Cg(1a)	4.047(3)	0	25.21	25.21	-3.662(2)	-3.662(2)
Cg(1)•••Cg(1b)	4.214(3)	0	29.10	29.10	3.682(2)	3.682(2)
			**2**			
Cg(1)•••Cg(1c)	3.7959(6)	0	15.66	15.66	3.6551(1)	-3.6551(1)
			**3**			
Cg(1)•••Cg(1d)	3.9058(16)	0	20.37	20.37	-3.6618(1)	3.6618(1)
			**5**			
Cg(1)•••Cg(1e)	3.9431(16)	0	23.03	23.03	3.6290(1)	- 3.6290(1)
			**8**			
Cg(1)•••Cg(2f)	3.9784(11)	0	22.52	22.52	-3.6750(1)	-3.6750(1)
			**10**			
Cg(3)•••Cg(4g)	3.836(2)	4.5(2)	17.19	21.62	3.5664(15)	-3.6651(19)
Cg(3)•••Cg(4h)	3.864(2)	4.5(2)	18.61	15.96	-3.7150(15)	3.6618(19)
Cg(5)[i]•••Cg(6i)	3.790(2)	0.6(2)	15.71	16.30	3.6375(16)	-3.6483(19)
			**11**			
Cg(3)•••Cg(6j)	3.7636(12)	7.26(10)	15.10	20.32	3.5294(7)	-3.6337(9)
Cg(3) •••Cg(6k)	3.8306(12)	7.26(10)	14.79	20.20	-3.5949(7)	3.7037(9)

[a]Symmetry code: (a) =–*x*, –*y*, 1–*z*; (b) =1–*x*, –*y*, 1–*z*; (c) = 3/2–*x*, –1/2+*y*, 3/2–*z*; (d) = 1/2–*x*, –1/2–*y*, 1–*z*; (e) = –1/2–*x*, –1/2+*y*, 2–*z*; (f) = 1/2–*x*, 1/2–*y*, *z*; (g) = –1+*x*, *y*, –1+z; (h) = *x*, *y*, –1+*z*; (i) = *x*, *y*, 1+z; (j) = 1/2–*x*, –1/2+*y*, 1/2–*z*; (k) = 1/2–*x*, 1/2+*y*, 1/2–*z*;

[b]α = dihedral angle between *Cg*(I) and *Cg*(J);

[c] β = angle *Cg*(I)→*Cg*(J) vector and normal to ring I;

[d] γ = angle between the *Cg*(I) →*Cg*(J) vector and the normal to plane J;

[e] *Cg*(I)-Perp = Perpendicular distance of *Cg*(I) on ring J;

[f]*Cg*(J)-Perp = perpendicular distance of *Cg*(J) on ring I

Cg1 is the centroid of the N(1)/C(1)/C(2)/N(2)/C(3)/C(4) set of atoms;

Cg2 is the centroid of the N(1)/C(1)/C(2)/N(2)/C(2a)/C(1a) set of atoms [(a) = *x*, 1–*y*, 1–*z*],;

Cg3 is the centroid of the N(1)/C(1)/C(2)/C(3)/N(2)/C(4) set of atoms;

Cg4 is the centroid of the C(15)/C(16)/C(17)/C(18)/C(19)/C(20) set of atoms;

Cg5 is the centroid of the N(3)/C(11)/C(12)/C(13)/N(4)/C(14) set of atoms;

Cg6 is the centroid of the C(5)/C(6)/C(7)/C(8)/C(9)/C(10) set of atoms.

Table S6. Short intra- and intermolecular contacts detected in structures **2, 4, 6** and **7, 9-13.**[a]

**D**	**A**	**D—H** [Å]	**H•••A** [Å]	**D•••A** [Å]	**D—H•••A** [°]
			**2**		
C(5)	N(4a)	0.93	2.57	3.353(3)	142.0
			**4**		
C(9)	N(96b)	0.93	2.60	3.291(4)	131.0

C(10)	O(2c)	0.96	2.42	3.298(5)	151.0
**6**					
O(98)	N(1)	0.85	1.99	2.835(3)	171.0
O(98)	N(97d)	0.85	2.03	2.877(3)	176.0
**7**					
O(1)	N(3c)	0.85	1.88	2.724(3)	167.3
C(5)	N(99)	0.96	2.40	3.271(5)	
**9**					
C(2)	N(4d)	0.93	2.59	3.457(3)	156.0
**10**					
C(4)	N(10)	0.93	2.62	3.125(5)	114.0
C(7)	N(6e)	0.93	2.53	3.326(6)	144.0
C(10)	N(2)	0.93	2.44	2.772(4)	101.0
C(20)	N(4)	0.93	2.50	2.822(4)	100.0
**11**					
N(3)	N(2f)	0.86	2.44	3.247(3)	158.0
N(3)	N(4)	0.86	2.62	3.217(3)	128.0
N(3)	N(6)	0.86	2.43	3.070(3)	132.0
C(1)	N(4)	0.93	2.57	3.089(3)	116.0
**12**					
N(3)	N(5g)	0.86	2.52	3.315(5)	154.0
C(2)	N(5g)	0.93	2.58	3.393(5)	146.0
**13**					
N(2)	N(3)	0.86	2.49	3.056(8)	124.0
N(2)	N(5h)	0.86	2.40	3.030(8	130.0
N(2)	N(4i)	0.86	2.17	3.024(9)	170.0

[a]Symmetry code: (a) = 2–*x*,–*y*,–*z*; (b) = 1–*x*, 1–*y*, *z*; (c) = *x*, 1–*y*, *z*; (d) = 1+*x*, *y*, *z*; (d) = 3/2–*x*, 3/2+*y*, 3/2–*z*; (e) = 1–*x*, –*y*, –*z*; (f) = 1/2–*x*, 1/2–*y*,1–*z*; (h) = –*x*, –*y*, –*z*; (i) = 1/2–*x*, –*y*, 1/2+z; (g) = 1/2–*x*, –1/2+*y*, 1–*z*;

Table S7. C—H•••Cg(J)(π-ring) interactions for **7** and **9**.[a]

C–X(I)•••Cg(J)	X(I)•••Cg(J) [Å]	X-Perp [Å]	γ [°][b]	C–X(I)•••Cg(J) [°]
**7**				
C(6)–H(6)•••Cg(1a)[c]	2.967	-3.7457	16.79	149.00
**9**				
C(5)–H(5)•••Cg(2b)[c]	2.872	3.7184(5)	13.94	148.00

[a]Symmetry codes: (a) = 1/2–*x*, –1/2+*y*, –*z*; (b) = 1/2–*x*, 1/2+*y*, 3/2–*z*
[b] γ = angle X(I)→Cg(J) vector and normal to plane J.
[c]Cg1 is the centroid of the N(1)/C(1)/C(2)/N(2)/C(3)/C(4) set of atoms;
Cg1 is the centroid of the N(1)/C(1)/C(2)/C(3)/ N(2)/C(4)

Table S8. Crystal data and structure refinement for **1-8**

	**1**	**2**	**3**	**4**	**5**	**6**	**7**	**8**
Empirical formula	$C_{16}H_{16}CoN_{10}$	$C_{12}H_6Cl_2CoN_{10}$	$C_{12}H_6CoI_2N_{10}$	$C_{32}H_{28}Co_3N_{26}O_4$	$C_{14}H_{10}N_{10}CoCl_2$	$C_{10}H_{12}CoN_8O_2$	$C_{36}H_{36}N_{26}O_2Co_3$	$C_{10}H_8CoN_8$
Formula weight	407.32	420.10	603.00	1017.59	448.15	335.21	1041.70	299.17
T, K	293.0(2)	293.0(2)	293.0(2)	293.0(2)	293.0(2)	293.0(2)	293.0(2)	293.0(2)
Wavelength, Å	0.71073	0.71073	0.71073	0.71073	0.71073	0.71073	0.71073	0.71073
Crystal system	Triclinic	Monoclinic	Monoclinic	Orthorhombic	Monoclinic	Triclinic	Monoclinic	Orthorhombic
Space group	$P\bar{1}$	*I*2/*m*	*C*2/*m*	*Pbam*	*C*2/*m*	$P\bar{1}$	*C*2/*m*	*Cmmm*
	a = 7.3512(8)	*a* = 9.2385(8)	*a* = 15.136(4)	*a* = 13.6519(5)	*a* = 9.3551(12)	*a* = 7.0909(13)	*a* = 19.0735(15)	*a* = 17.2970(18)
	b = 8.5713(14)	*b* = 7.3102(6)	*b* = 7.3235(16)	*b* = 21.1794(7)	*b* = 7.2579(8)	*b* = 7.1913(14)	*b* = 7.4913(8)	*b* = 7.3500(9)
Unit cell dimensions,	*c* = 9.0308(9)	*c* = 12.4932(13)	*c* = 9.6109(17)	*c* = 7.3175(3)	*c* = 16.0861(19)	*c* = 8.071(15)	*c* = 15.7840(11)	*c* = 9.7480(11)
Å and deg	α = 93.343(11)					α = 82.427(16)		
	β = 113.229(10)	β = 95.708(9)	β = 122.992(15)		β = 121.203(8)	β = 86.566(15)	β = 92.501(7)	
	γ = 113.730(13)					γ = 61.69(2)		
V, Å^3	462.48(11)	839.56(14)	893.6(4)	2115.9(13)	934.2(2)	359.15(13)	2253.1(3)	1239.3(2)
Z	1	2	2	2	2	1	2	4
D_c, g cm^{-3}	1.462	1.662	2.241	1.597	1.593	1.550	1.535	1.603
Absorption coefficient, mm^{-1}	0.951	1.359	4.433	1.234	1.227	1.212	1.158	1.384
F(000)	209.0	418.0	562.0	1030.0	450.0	171.0	1062.0	604.0
Crystal size, mm	0.08×0.07×0.04	0.66×0.15×0.07	0.17×0.11×0.05	0.08×0.04×0.04	0.16×0.08×0.03	0.20×0.06×0.03	0.16×0.06×0.05	0.13×0.05×0.02
θ range for data collection [°]	3.40 to 25.05	3.56 to 25.05	3.50 to 29.33	3.302 to 25.04	3.56 to 29.57	3.33 to 29.45	3.42 to 25.05	3.67 to 29.55
	$-8 \le h \le 8$	$-11 \le h \le 10$	$-19 \le h \le 20$	$-16 \le h \le 16$	$-21 \le h \le 22$	$-9 \le h \le 7$	$-22 \le h \le 22$	$-23 \le h \le 19$
Index ranges	$-10 \le k \le 9$	$-8 \le k \le 8$	$-9 \le k \le 9$	$-25 \le k \le 25$	$-10 \le k \le 9$	$-9 \le k \le 7$	$-8 \le k \le 8$	$-7 \le k \le 9$
	$-10 \le l \le 10$	$-14 \le l \le 14$	$-12 \le l \le 12$	$-8 \le l \le 7$	$-12 \le l \le 11$	$-10 \le l \le 10$	$-18 \le l \le 18$	$-13 \le l \le 11$
Reflections collected	3826	2705	2766	11013	4531	3499	8357	3594
Independent reflections	1642 [R_{int} =0.0413]	798 [R_{int} = 0.0244]	1155 [R_{int} =0.0457]	2032 [R_{int} =0.0328]	1251 [R_{int} =0.0281]	1700 [R_{int} =0.0520]	2148 [R_{int} =0.0500]	910 [R_{int} = 0.0458]
Min. and max. transm.	0.939 and 1.000	0.493 and 1.000	0.348 and 1.000	0.854 and 1.000	0.598 and 1.000	0.855 and 1.000	0.489 and 1.000	0.624 and 1.000
Data/restraints/parameters	1642/ 0/ 125	798 / 0 / 71	1155/ 0/ 71	2032/ 0/ 180	1251/0/78	1700/ 0/ 99	218/ 0/ 177	910/ 0/ 66
Goodness-of-fit on F^2	1.077	1.124	1.109	1.086	1.104	1.054	1.090	1.128

Final R indices [$I > 2\sigma(I)$]	R_1 = 0.0530<br>wR_2 = 0.1283	R_1 = 0.0259<br>wR_2 = 0.0663	R_1 = 0.0351<br>wR_2 = 0.0872	R_1 = 0.0281<br>wR_2 = 0.0714	R_1 = 0.0712<br>wR_2 = 0.2229	R_1 = 0.0439<br>wR_2 = 0.0752	R_1 = 0.0446<br>wR_2 = 0.1124	R_1 = 0.0443<br>wR_2 = 0.1160
R indices (all data)	R_1 = 0.0606<br>wR_2 = 0.1351	R_1 = 0.0285<br>wR_2 = 0.0676	R_1 = 0.0460<br>wR_2 = 0.0937	R_1 = 0.0358<br>wR_2 = 0.0746	R_1 = 0.0792<br>wR_2 = 0.2303	R_1 = 0.0645<br>wR_2 = 0.0929	R_1 = 0.0603<br>wR_2 = 0.1185	R_1 = 0.0526<br>wR_2 = 0.1215
Largest diff. peak/hole, e $Å^{-3}$	1.49/–0.40	0.20/–0.32	1.24/–0.66	0.27/–0.38	1.51/–1.01	0.46/–0.67	0.75/–0.56	1.18/–0.64
CCDC number	2482673	1867785	2482671	2482672	2482670	2482656	2482661	2482674

Table S9. Crystal data and structure refinement for **9–13**.

	**9**	**10**	**11**	**12**	**13**
Empirical formula	$C_{14}H_{12}CoN_{10}$	$C_{24}H_{16}CoN_{10}$	$C_{24}H_{18}CoN_{12}$	$C_{12}H_{10}CoN_{12}$	$C_8H_4CoN_9$
Formula weight	379.27	503.40	533.43	381.25	285.13
T, K	293.0(2)	295.0(2)	295.0(2)	295.0(2)	295.0(2)
Wavelength, Å	0.71073	0.71073	0.71073	0.71073	0.71073
Crystal system	Monoclinic	Triclinic	Monoclinic	Monoclinic	Orthorhombic
Space group	$I2/m$	$P\bar{1}$	$C2/c$	$C2/m$	$Pnma$
	a = 6.2797(5)	a = 7.3667(4)	a = 24.3451(11)	a = 18.8324(13)	a = 12.8414(6)
	b = 7.3851(5)	b= 12.4526(5)	b = 7.4382(3)	b= 7.3295(4)	b= 11.7288(7)
Unit cell dimensions,	c = 17.9764(12)	c = 127968(5)	c = 15.0196(7)	c = 6.1774(4)	c = 9.6094(5)
Å and deg		α = 92.385(3)			
	β = 92.969	β = 104.108(4)	β = 122.102(3)	β = 107.311(6)	
		γ = 94.807(4)			
V, Å^3	832.55	1132.16(9)	2303.95(19)	814.06(9)	1447.31(14)
Z	2	2	4	2	4
D_c, g cm^{-3}	1.513	0.794	1.538	1.555	1.309
Absorption coefficient, mm^{-1}	1.051	1.477	0.787	1.078	1.183
F(000)	386.0	514.0	1092.0	386.0	568.0
Crystal size, mm	0.280×0.150×0.120	0.140×0.130×0.080	0.120×0.120×0.09	0.280×0.120×0.060	0.240×0.08×0.07
θ range for data collection [°]	3.50 to 29.24	3.27 to 25.05	3.52 to 25.04	3.45 to 25.03	3.47 to 25.49
Index ranges	-7 ≤ h ≤ 7	-7 ≤ h ≤ 8	-26 ≤ h ≤ 28	-19 ≤ h ≤ 22	-15 ≤ h ≤ 15
	-9 ≤ k ≤ 10	-14 ≤ k ≤ 14	-8 ≤ k ≤ 8	-8 ≤ k ≤ 8	-10 ≤ k ≤ 14
	-23 ≤ l ≤ 24	-15 ≤ l ≤ 15	-17 ≤ l ≤ 15	-7 ≤ l ≤ 7	-9 ≤ l ≤ 11
Reflections collected	4275	11157	8730	3213	6781
Independent reflections	1107 [R_{int} = 0.0244]	4003 [R_{int} = 0.0336]	2029 [R_{int} = 0.0279]	784 [R_{int} = 0.0314]	1407 [R_{int} = 0.0284]
Min. and max. transm.					
Data / restraints / parameters	1107/0/72	4003/0/316	2029/0/168	784/0/71	1407/0/91
Goodness-of-fit on F^2	1.095	1.113	1.088	1.158	1.097

Final R indices [$I > 2\sigma(I)$]	R_1 = 0.0262<br>wR_2 = 0.0611	R_1 = 0.0469<br>wR_2 = 0.1212	R_1 = 0.0307<br>wR_2 = 0.0786	R_1 = 0.0280<br>wR_2 = 0.0733	R_1 = 0.0612<br>wR_2 = 0.2213
R indices (all data)	R_1 = 0.0303<br>wR_2 = 0.0634	R_1 = 0.0580<br>wR_2 = 0.1259	R_1 = 0.0355<br>wR_2 = 0.0804	R_1 = 0.0299<br>wR_2 = 0.0744	R_1 = 0.0741<br>wR_2 = 0.2346
Largest diff. peak and hole, e Å^{-3}	0.29 and –0.20	0.72 and –0.35	0.39 and –0.22	0.33 to –0.36	1.68 and –0.54
CCDC number	2482657	2482659	2482662	2482658	2482660
